# Multimodal single-cell profiling reveals crosstalk between macrophages and stromal cells in poor prognostic cholangiocarcinoma patients

**DOI:** 10.1038/s41698-026-01292-6

**Published:** 2026-01-28

**Authors:** Lara Heij, Sikander Hayat, Konrad Reichel, Sidrah Maryam, Colm J. O’Rourke, Xiuxiang Tan, Marlous van den Braber, Jan Verhoeff, Maurice Halder, Fabian Peisker, Georg Wiltberger, Jan Bednarsch, Daniel Heise, Julia Campello Deierl, Sven A. Lang, Florian Ulmer, Tom Luedde, Edgar Dahl, Danny Jonigk, Jochen Nolting, Shivan Sivakumar, Jens Siveke, Florian Vondran, Flavio G. Rocha, Hideo A. Baba, Sylvia Hartmann, Jesper B. Andersen, Zaynab Hobloss, Ahmed Ghallab, Jan G. Hengstler, Juan J. Garcia Vallejo, Rafael Kramann, Ulf Neumann

**Affiliations:** 1 Department of Surgery and Transplantation, University Hospital Essen, Essen, Germany; 2 Department of Pathology, University Hospital Essen, Essen, Germany; 3 Department of Renal and Hypertensive Disorders, Rheumatological and Immunological Diseases (Medical Clinic II), Medical Faculty, RWTH Aachen University, Aachen, Germany; 4 Department of Pathology, Erasmus MC, University Medical Center Rotterdam, Roterdam, The Netherlands; 5 Cardiovascular Research Institute and Department of Medicine, Icahn School of Medicine at Mount Sinai, New York, USA; 6 Amsterdam UMC – Location Vrije Universiteit Amsterdam, Molecular Cell Biology & Immunology, Amsterdam, The Netherlands; 7 NUTRIM School of Nutrition and Translational Research in Metabolism, Maastricht University, Maastricht, The Netherlands; 8 Department of Health and Medical Sciences, Biotech Research and Innovation Centre (BRIC), University of Copenhagen, Copenhagen, Denmark; 9 Department of Surgery and Transplantation, University Hospital RWTH Aachen, Aachen, Germany; 10 Amsterdam Infection & Immunity, Cancer Immunology, Amsterdam, The Netherlands; 11 Tytgat Institute for Liver and Intestinal Research, Amsterdam UMC, University of Amsterdam, Amsterdam, the Netherlands; 12 Department of Surgery, Maastricht University Medical Center (MUMC), Maastricht, The Netherlands; 13 Department of Gastroenterology, Hepatology and Infectious Diseases, University Hospital Duesseldorf, Düsseldorf, Germany; 14 Institute of Pathology, University Hospital RWTH Aachen, Aachen, Germany; 15 Center for Integrated Oncology Aachen Bonn Cologne Duesseldorf (CIO ABCD), Aachen, Germany; 16 Biomedical Research in Endstage and Obstructive Lung Disease Hannover (BREATH), German Center for Lung Research (DZL), Hannover, Germany; 17 Institute of Pathology, Hannover Medical School, Hannover, Germany; 18 University of Birmingham and University hospitals of Birmingham NHS Trust, Birmingham, UK; 19 Bridge Institute of Experimental Tumor Therapy, West German Cancer Center, University Hospital Essen, University Duisburg-Essen, Essen, Germany; 20 Division of Surgical Oncology, Knight Cancer Institute, Oregon Health and Science University, Portland, OR USA; 21 Department of Toxicology, Leibniz Research Centre for Working Environment and Human Factors, Technical University Dortmund, Dortmund, Germany; 22 Department of Forensic Medicine and Toxicology, Faculty of Veterinary Medicine, South Valley University, Qena, Egypt

**Keywords:** Cancer, Computational biology and bioinformatics, Immunology, Oncology

## Abstract

Cholangiocarcinoma (CCA) is a deadly cancer, characterized by abundant stroma. The tumor microenvironment (TME) plays an important role in its aggressive behavior and poor response to therapeutics; however, the underlying pathways are unknown. To fill this gap, we used multiplexed immunohistochemistry, high-dimensional cytometry, and single cell transcriptomics. Our findings confirm an abundance of regulatory T cells (Tregs) and a lack of effector memory T cells within the tumor. Tumor-infiltrating T cells show signs of exhaustion. Using our transcriptomic data, we revealed cellular crosstalk in poor prognosis patients. This crosstalk is driven by stromal cells and macrophages. Among the responsible receptor-ligand pairs are GAS6-AXL, VCAN-TLR2, and EGFR-TGF-β. The multiple mechanisms leading to the exclusion of relevant immune cells needed for an anti-cancer response and mechanisms leading to active immune suppression are part of complex cell-cell crosstalk. This study provides a deeper insight into the immune exhausted phenotype in CCA.

## Introduction

Cholangiocarcinoma (CCA) is a deadly disease occurring along the biliary tract and is considered a rare cancer type with a 5-year overall survival (OS) rate of 7–20%^1,2^. CCA is classified as intrahepatic cholangiocarcinoma (iCCA), perihilar cholangiocarcinoma (pCCA), or distal cholangiocarcinoma (dCCA). Although the histological pattern can be very similar, the subtypes have been considered different entities because of differences in their surrounding tissue and molecular tumor heterogeneity^3–5^. CCA typically shows distinctive perineural invasion (PNI), defined as the invasion of cancer cells into and around the nerve trunks. Recently, we showed that both iCCA and pCCA may present a high nerve fiber density (NFD) in the tumor microenvironment (TME), which is linked to better survival in a subset of patients^6,7^. Another shared histological feature of CCA subtypes is the presence of an abundant stromal compartment. The stroma displays heterogeneous cell types, including immune cells and fibroblasts. Interactions between different cell types result in immune-infiltrated or immune-excluded phenotypes^8–10^.

Recent progress in cancer care has included the development of immune checkpoint inhibitors (ICI) and therapeutic agents that lead to host immune cell recruitment to target cancer. Specifically, T cells play an important role in the immune response of the host to cancer and are the main factor leading to curative responses to checkpoint inhibitor immunotherapy. In CCA, regulatory T cells (Tregs) dominate the early immune response and remain present in later tumor stages^11,12^, whereas effector T cells are rarely found, and if present, do not show signs of activation^13^. The presence of CD4 and CD8 T cells infiltrating the tumor and peritumoral areas is a sign of better outcomes^14^, and high numbers of CD8 T cells in the outer border of the tumor are positively correlated with 5-year survival rates in patients with iCCA^15^. Furthermore, a subset of CD8 T cells expressing CD161 is known to have higher cytotoxic potential^16,17^, and high levels of CD161 expression are associated with protective functions in many cancer types, including CCA^18^. The immune checkpoint PD-1 is known to promote immune evasion, and high PD-1 levels in tumor-infiltrating CD8 T cells in iCCA were previously found to be associated with an unfavorable prognosis^19^. Nevertheless, our understanding of the immune cell environment in CCA remains limited, and additional factors related to treatment responses are still needed^20^.

In addition to T cells, tumor-associated macrophages (TAMs) constitute another major immune population within the TME. Although TAMs can exhibit both pro- and anti-inflammatory functions, they are thought to be mostly immunosuppressive^21^. Despite some disagreement, most studies point to a worse prognosis in patients with high levels of TAMs^22–24^. TAMs are a major source of PD-1 ligand (PD-L1) in CCA tumors^25^. Furthermore, TAMs can secrete cytokines, such as tumor growth factor-β (TGF-β), which suppresses the T cell response by T cell exclusion and prevents the acquisition of the tumor-suppressive TH1-effector phenotype^26^. Despite its immune-regulatory functions, modulation of the extracellular matrix (ECM) plays a crucial role in recruiting TAMs in tumors^27^.

In this study, we aimed to better characterize the stromal compartment in CCA through a comprehensive approach using cytometry by time-of-flight (CyTOF) and single nuclei (sn)RNAseq. We focused on patients with primary resected iCCA and pCCA and characterized the differences in immune landscapes of both entities in primary tumors, compared to matched peripheral liver tissue, and peripheral blood samples. To identify the crosstalk between cell clusters responsible for the immunosuppressive environment, we used snRNA sequencing to characterize the cellular heterogeneity of the transcriptome in patients with pCCA. We identified signs of exhaustion and an increased abundance of regulatory T cells in the tumor niche, while CD8 effector memory (EM) and tissue-resident effector memory cells (TRMs) failed to reach the tumor and were restricted to the peripheral liver tissue. To further explore the immunosuppressive TME, we revealed a shift in crosstalk between stromal cells and specific cancer cells, mainly present in the poor prognosis group. In addition, these cancer cells demonstrated an upregulated cell communication with macrophage-myeloid cells. Overall, this study sheds light on the etiology of immunosuppressive TME in CCA.

## Results

**Fig. 1:**
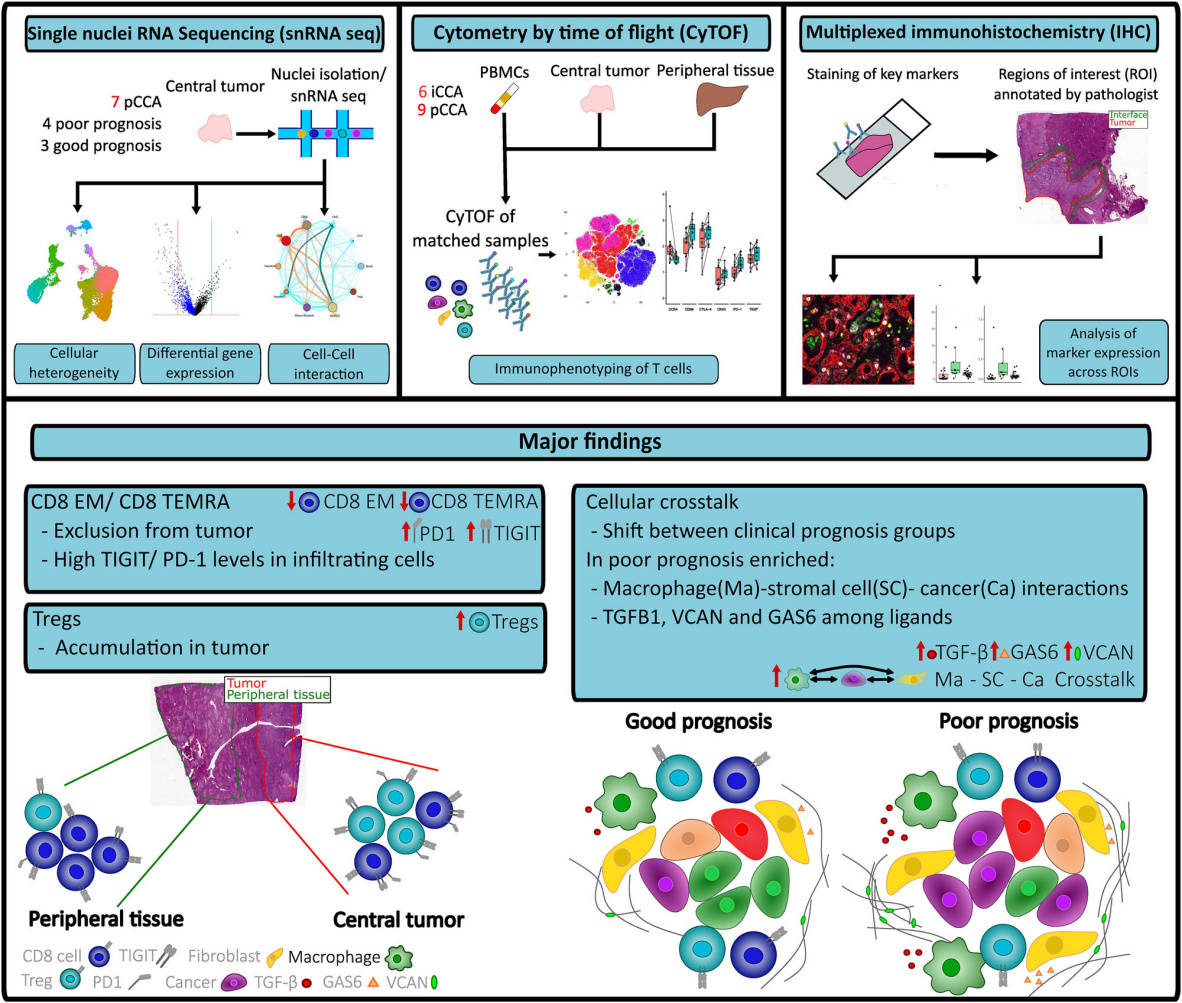
Study workflow and overview of results. In our study, we included patients with primary intrahepatic cholangiocarcinoma (iCCA) or perihilar cholangiocarcinoma (pCCA) scheduled for resection. To examine the tumor microenvironment (TME) we used single nuclei RNA sequencing (snRNAseq), Cytometry by time of flight (CyTOF) and multiplexed immunohistochemistry (IHC). For snRNAseq, we analyzed 7 pCCA samples grouped into poor prognosis (*n* = 4) and good prognosis (*n* = 3) groups based on clinical data. For CyTOF we used matched blood, tumor, and peripheral samples from 6 iCCA and 9 pCCA patients. To gain further spatial information we used IHC data where a pathologist annotated tumor/ interface regions. Combining the data, we were able to identify an exclusion of CD8 T cell subsets from the tumor and an accumulation of regulatory T cells (Tregs) in the tumor. Tumor-infiltrating T cells expressed high levels of several immune checkpoints. We also revealed a shift in the cell-cell interactions in the poor prognosis patients. This shift was marked by increased interactions between macrophages, stromal cells, and cancer cells. Enriched ligands included VCAN, TGFB1, and GAS6.

To elucidate the heterogeneity of the TME, we performed high-dimensional immunophenotyping using mass cytometry time-of-flight (CyTOF) on 16 tumor samples. Of these, seven were iCCA patients and nine were pCCA patients with resectable disease. For all patients, we obtained matched tumor-free liver tissue (hereafter referred to as ‘peripheral tissue’), central tumor tissue, and peripheral blood samples (Fig. 1). All patients underwent surgery at the University Hospital RWTH Aachen to obtain detailed patient characteristics see Table 1. Mass cytometry was performed using the panel described in Supplementary Table 1, with a strong emphasis on the characterization of different subsets of T cells and the expression of known costimulatory molecules and immune regulators of importance in cancer immunotherapy. This was later combined with immunohistochemistry (IHC) to give more spatial context and results from snRNA-seq to gain a more complete understanding of the TME (Fig. 1).

**Table 1:** Clinical data for patients included in the CyTOF cohort

	iCCA	pCCA		
**Included patients**	6	9		
**Age in years**	68.7	64.2		
**RFS in months**	17.2	11.8		
**Survival time in months**	22.8	14.2		
**Grade**				
G1	0	0%	1	11%
G2	3	50%	5	56%
G3	2	33%	3	33%
G4	0	0%	0	0%
not known	1	17%	0	0%
**Perineural invasion**				
positive	2	33%	1	11%
negative	3	50%	8	89%
not known	1	17%	0	0%
**Lymph node invasion**				
positive	4	67%	3	33%
negative	1	17%	6	67%
not known	1	17%	0	0%
**Lymphovascular invasion**				
positive	0	0%	2	22%
negative	6	100%	7	78%
not known	0	0%	0	0%

First, we used a combination of unsupervised clustering and manual gating to identify T cells in the CyTOF data and focused on the analysis of CD4 and CD8 cells (Supplementary Fig. S1). Downstream analysis of both compartments using a combination of dimensionality reduction (UMAP) and clustering (Phenograph) resulted in the identification of 27 CD4 T-cell clusters and 31 CD8 T-cell clusters, which were manually curated and annotated into known immune cell subsets (Supplementary Fig. S2). For comparison we also performed manual gating of the same subset (Supplementary Fig. S3).

### The spatial distribution of CD4 and CD8 memory cells differs between peripheral tissue and tumor tissue, but not between tumor entities

**Fig. 2:**
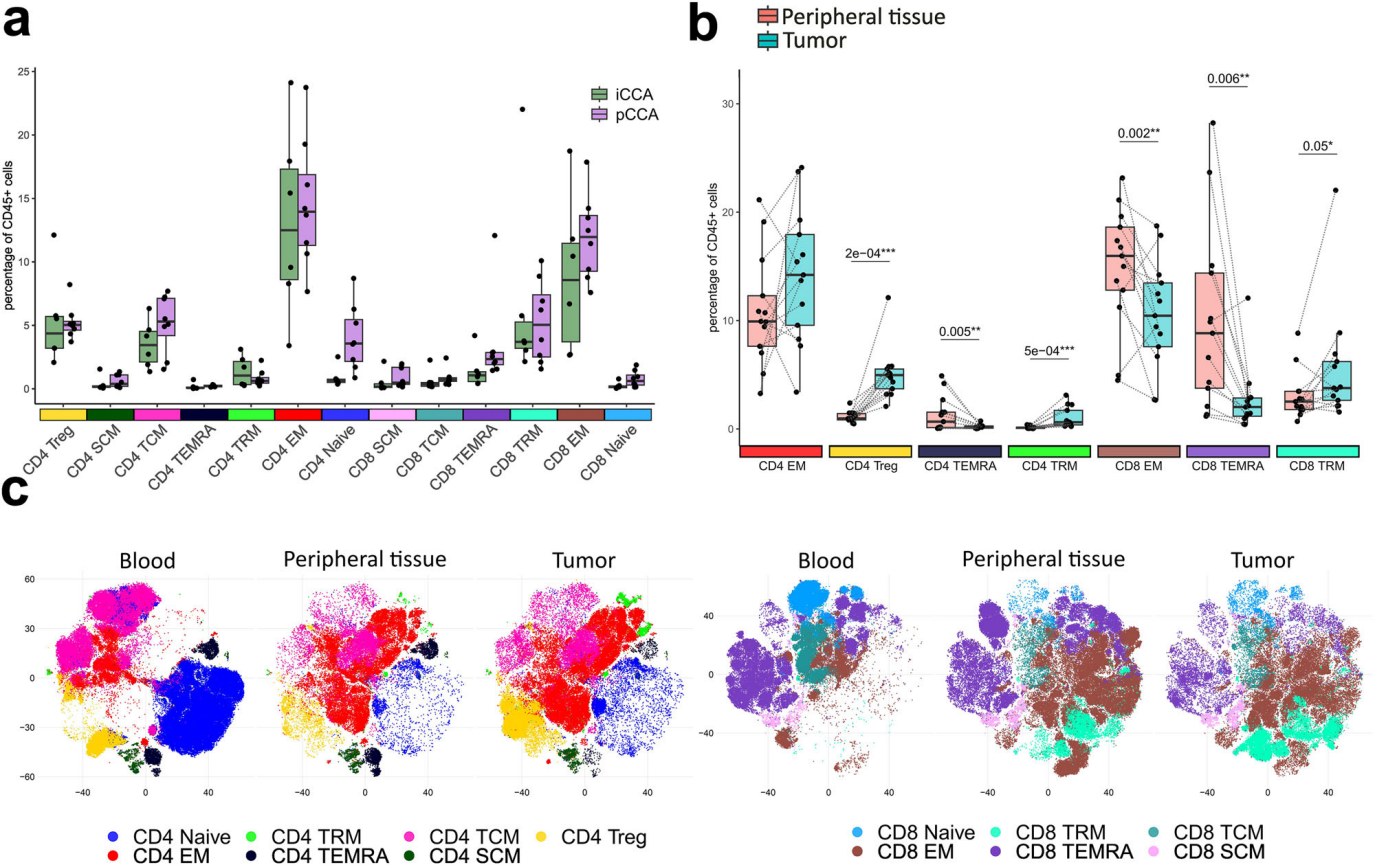
High-dimensional phenotyping in iCCA. **a** Mean percentage of T cell subsets of total viable CD45+ cell types in the tumor. Annotation was done by manual annotation of clusters after unsupervised clustering. No subset is significantly enriched in one of the two CCA subtypes. **b** The mean percentage of T cell subsets of total viable CD45+ cell types in matched peripheral tissue and tumor samples shows enrichment of Tregs and exclusion of CD8 EM and CD8 TEMRAs from the tumor. Shown p values were obtained using the Wilcoxon test and were adjusted for multiple testing. **c** Dimensionality reduction plots (optSNE) of CD4 and CD8 T cells from all patients reveal heterogeneity within different sample locations. Split by origin tissue for CD4 cells (left) and CD8 cells (right).

Using an unsupervised clustering-based approach, we uncovered differences between the CD4 and CD8 memory compartments. Notably, the abundance of none of the examined subsets was significantly elevated when comparing the iCCA and pCCA tumor samples (Fig. 2a). This suggests a comparable T-cell composition in the iCCA and pCCA subtypes, which enabled a combined analysis of both entities. Looking at cell abundance between the matched tumor and peripheral tissue, we observed a 5-fold increase in CD4 Tregs (*p* = 0.0002) and an increase in CD4 tissue-resident memory cells in the tumor tissue (*p* = 0.0005). In the CD8 compartment, CD8 effector memory and tissue-resident effector memory RA cells (TEMRA) were more abundant (*p* = 0.002 and *p* = 0.006, respectively) in the peripheral tissue, and CD8 tissue-resident memory cell levels were elevated in the tumor tissue (*p* = 0.05) (Fig. 2b). Besides the differences in subset abundance, the low-dimensional representation revealed a more complex heterogeneity within most subsets (Fig. 2c). For the abovementioned comparisons, we observed the same trends when analyzing iCCA and pCCA separately. Although some of the changes do not reach significance in both entities (Supplementary Table 2, Supplementary Figs. S4 and S5).

### Exhausted effector cells in the tumor niche with less cytotoxic potential

**Fig. 3:**
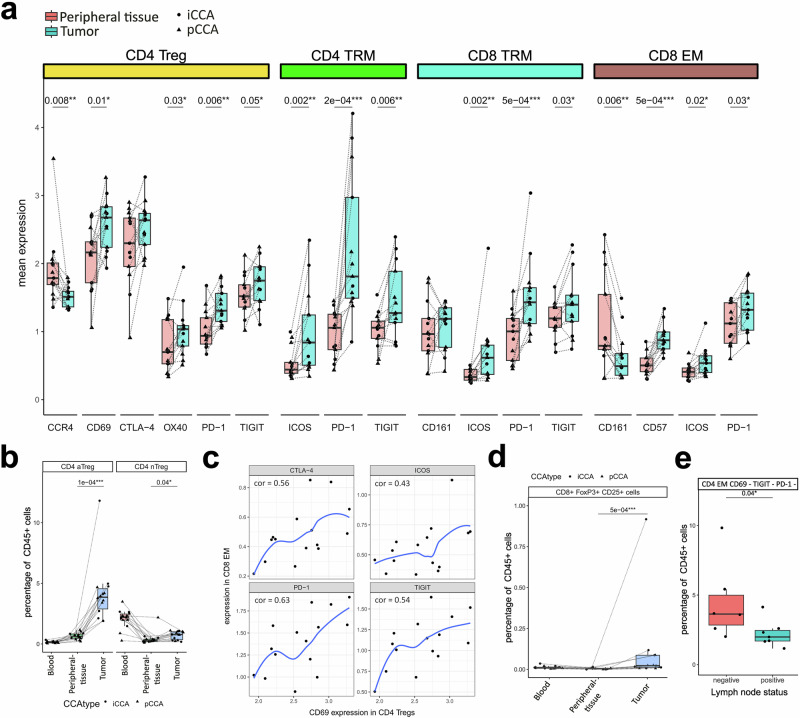
Characterization of marker expression in cell clusters. **a** Mean normalized expression of markers across CD4 Treg, CD8 TRM, and CD8 EM cells shows an increased expression of checkpoints/ activations markers in the tumor. **b** A specific cluster of T cells (aTreg = activate Treg) with high levels of activation markers is enriched in the tumor but is absent from the blood. Tregs lacking activation markers are also enriched in the tumor but are also common in the blood (nTreg = non-activated Treg). **c** Mean checkpoint marker expression in CD8 EM cells correlates with mean CD69 expression in the Treg population within the tumor. The correlation coefficient was calculated using spearman and shown in blue is a regression line. **d** CD25 and FoxP3 positive CD8 cells were identified in the tumor by sequential gating. Shown is the mean percentage of total CD45 positive live cells per sample. **e** One cluster of CD4 EM cells lacking expression of activation markers and checkpoints is enriched in the peripheral tissue of CCA patients without cancer cell invasion of adjacent lymph nodes.

In addition to the sparseness of CD8 effector memory (EM) and TEMRA cells in the tumor, CD8 EM showed a decrease in CD161 expression compared to the peripheral tissue, indicating less cytotoxic activity (Fig. 3a). In addition, many of the identified memory subtypes display higher levels of immune checkpoints in tumors. While PD-1 and ICOS levels were elevated in CD8 EMs, CD8 TRMs, CD4 TRMs, and CD8 TRMs, the CD4 TRMs also exhibited higher levels of TIGIT. In addition to the elevated levels of immune checkpoints, we also observed an increase in CD57 expression. CD57 is used as a marker for differentiation/senescence and may promote resistance to ICI in cancer^28,29^. Furthermore, the marker expression profile in the small subset of CD4 stem cell memory (SCM) also showed signs of overactivation in tumors with elevated levels of HLA-DR (*p* < 0.01), CTLA-4 (*p* < 0.01), and ICOS (*p* < 0.05).

### Activated regulatory T cells correlate with exhausted CD8 effector memory cells

CD4 Tregs showed elevated levels of the activation markers CD69 and OX40, and the immune checkpoints PD-1, CTLA-4, and TIGIT. The chemokine receptor CXCR3 (p value did not reach significance) and CCR4 showed decreased levels in the tumor. Both receptors are associated with the migration to tumor tissue^30^. Within the total Treg population, we identified a distinct cluster of Tregs with lower levels of CCR7 and higher levels of CD69, indicating a more active state (Supplementary Fig. S6a). These activated Tregs (aTregs) were almost completely absent in the blood but were more than four times more abundant (*p* = 0.0001) in the tumor than in the peripheral tissue (Fig. 3b). Furthermore, the expression of CD69 by Tregs in the tumor tissue was positively correlated with the mean expression of CTLA-4, TIGIT, ICOS, and PD-1 in CD8 effector memory cells, indicating a connection between immune checkpoint expression in effector cells and the activation status of regulatory T cells (Fig. 3c). In addition to the well-studied CD4 Tregs, there was also an increase in CD8+ FoxP3 + CD25+ cells in the TILs population (Fig. 3d). Although this population is considerably smaller, it is thought to have regulatory functions similar to those of CD4 Tregs and is more frequent in some types of cancers^31,32^.

### Lymph node-negative patients demonstrate enrichment of CD4 EM cells lacking multiple checkpoint co-expression

To identify the aggressive biology of the disease, we looked at immune cell distribution in lymph node-negative patients. We observed a cluster of CD4 EM cells enriched in the peripheral liver tissue of patients without lymph node invasion (Fig. 3e). These CD4 EM cells are characterized by a lack of the expression of checkpoints CTLA-4, PD-1, and TIGIT as well as the activation marker CD69 (Supplementary Fig. S6b). There was no correlation with overall survival, most likely due to the small cohort size and high postoperative mortality rate of almost 25%.

### PD-1 positive CD4 and CD8 residential effector memory cells are present in 24% of our patient cohort

**Fig. 4:**
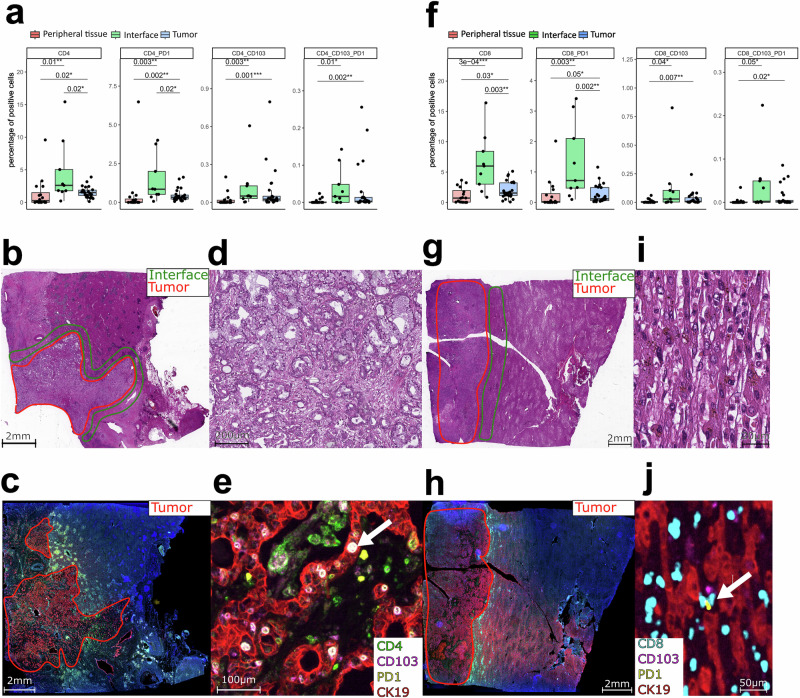
Validation by multiplexed immunohistochemistry. **a** Percentage of cells positive for CD4, CD103, and PD-1 in the different regions of interest (ROIs). PD-1/ CD103 positive cells were mainly found in the interface and tumor area, while positive cells for all markers were rare in the periphery (**b**) Overview of the HE-stained slide of patient 323. **c** The corresponding multiplexed image. **d** Magnified image of the HE-stained slide of the tumor area of patient 323. **e** The corresponding magnified multiplexed image. The highlighted cells with a box are CD4 effector memory cells with expression of PD-1. **f** Percentage of cells positive for CD8, CD103, and PD-1 in the different ROIs. Cells positive for the different markers were more abundant in the tumor and interface ROIs. **g** Overview of the HE-stained slide of patient 302. **h** The corresponding multiplexed image. **i** Magnified image of the HE-stained slide of the interface area of patient 302. **j** The corresponding magnified multiplexed image. The highlighted cells with a box are CD8 effector memory cells with expression of PD-1.

PD-1 expression in effector memory cells is a potential biomarker to allow patient stratification for PD-1 blockade therapy. We applied multiplexed immunohistochemistry (IHC) on whole slide images to identify the spatial distribution of CD4 and CD8 T cells including CD103 as a marker for tissue-resident memory cells (TRMs), combined with PD-1 expression. CD4 and CD8 cells were mainly present in the tumor and interface areas, with and without PD-1 co-expression. The regions annotated by the pathologist as peripheral liver tissue showed lower levels of all the examined CD4 subsets (Fig. 4a–j). Levels of PD-1 positive CD8 cells and CD8 TRMs were also more abundant in the tumor (T) and interface (I) regions compared to the peripheral tissue (P) (*p* = 0.003 (I-P) for CD8_PD-1 and *p* = 0.05 (T-P), *p* = 0.05 for CD8 TRM_PD1 (I-P) and *p* = 0.02 (T-P)) (Fig. 4f–j). These findings are in line with our CyTOF data, which demonstrated increased PD-1 expression in CD4 and CD8 TRMs in the tumor tissue. Also, CD4 and CD8 as well as PD-1 positive CD4 and CD8 cells were more abundant in the interface compared to the tumor region (*p* = 0.02 for CD4, *p* = 0.003 for CD8, *p* = 0.02 for CD4_PD-1, and *p* = 0.002 CD8_PD-1). In our cohort, 24% of the patients (6 of 25 patients) demonstrated either CD4 or CD8 TRM cells positive for PD-1, identifying a potential subgroup of patients who could respond to PD-1 blockade therapy. Of course, this finding would need further validation before this combination of markers could be used in the clinical field. Multiplexed immunohistochemistry is available in most university hospitals and could be a useful tool in the future since the spatiality of the immune cells is kept and plays a role.

### Transcriptomics reveals heterogeneity in cholangiocarcinoma cancer cell and stromal populations

To understand cellular interactions between different cell types we applied transcriptomics. We performed snRNA sequencing of tumor tissue from seven patients with pCCA, split into two groups based on their clinical prognosis. Patients with cancer-positive lymph nodes and cancer recurrence within one year after surgery were identified as having a poor prognosis based on the aggressive biology of the disease (*n* = 3 with good clinical prognosis; *n* = 4 with poor clinical prognosis). Detailed patient characteristics are outlined in Table 2, and available detected genetic variants are outlined in Supplementary Table 3. For snRNA sequencing, we were limited in the number of patients we were able to analyze and decided to focus on pCCA. ICCA is genetically distinct and diverse and would introduce further heterogeneity into the dataset, potentially masking underlying mechanisms^33^.

**Table 2:** Clinical data for patients included in snRNA sequencing

	Poor	Good		
**Included patients**	4	3		
**Age in years**	55.3	69.8		
**RFS in months**	8	17.7		
**Survival time in months**	12.7	17.7		
**Grade**				
G1	0	0%	1	33%
G2	0	0%	1	33%
G3	3	75%	1	33%
G4	0	0%	0	0%
not known	1	25%	0	0%
**Perineural invasion**				
positive	1	25%	0	0%
negative	2	50%	3	100%
not known	1	25%	0	0%
**Lymph node invasion**				
positive	4	100%	0	0
negative	0	0%	3	100%
not known	0	0%	0	0%
**Lymphatic or vascular invasion**				
positive	0	0%	1	33%
negative	3	75%	2	67%
not known	1	25%	0	0%

**Fig. 5:**
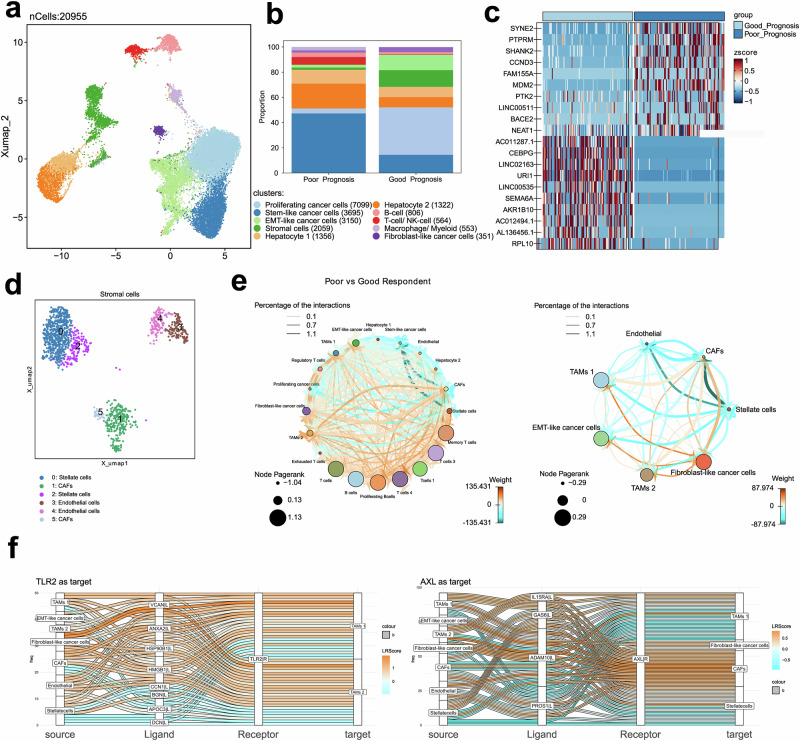
SnRNA reveals heterogeneity in cell clusters between clinical groups. **a** UMAP representation of clusters in single-cell data from 7 pCCA patients. **b** Stacked bar plot representing the compositional distribution of different cell types in good and poor prognosis. **c** Heat map plot representing top 10 differentially expressed up and down-regulated genes in poor prognosis taking good prognosis as a reference and all cancer cells taken together. **d** UMAP representation of sub-clustering within the stroma subset. Stromal cells were annotated based on marker gene expression into Stellate cells, Cancer associated fibroblasts (CAFs) and Endothelial cells. **e** Network graph depicting comparative cell-cell communication based on good and poor prognosis, between different cell types. Here, the good prognosis is taken as a reference, and higher weight defines up-regulated communication in disease as compared to control. On left interactions between all identified subsets are shown on the right we only show the subsets most involved in cell-cell-communication. **f** Sankey plots visualize the top ligand-receptor pairs within the most involved subsets. Shown are predicted interaction with TLR2 (left) and AXL (right) as target genes.

We identified 10 distinct clusters of cells (Fig. 5a), distributed across the two prognosis groups. These clusters were manually annotated into known cell types based on marker gene expression. A large proportion of cells, represented by 4 of the 10 identified clusters, expressed markers associated with cholangiocytes and malignant epithelial cells and were annotated as cancer cells. Other identified cell clusters included T cells, B cells, and a cluster of macrophages as three distinct immune cell populations. Two clusters of hepatocytes and a cluster of stromal cells were identified (Supplementary Fig. S7a). The different clusters were heterogeneously distributed between the patient and prognosis groups (Fig. 5b). Hepatocytes were mainly present in only two patients, and the proportion of cancer cell clusters also differed between patients (Supplementary Fig. 4b and 4c). For the total cancer cell population, we identified 4167 differentially upregulated and 5578 differentially downregulated genes (FDR < 0.05) between prognosis groups, including MDM2 and PTPRM (Fig. 5c).

The 4 identified cancer clusters were enriched in genes like MDM2, PTRM, PLCG2and RAP1B known to be involved in cancer development. Based on their gene expression, they were annotated into proliferating cancer cells, stem-like cancer cells, EMT-like cancer cells and Fibroblast cancer cells. (Supplementary Fig. S7b). To further examine the stromal compartment, we performed sub-clustering and identified Stellate cells, Endothelial cells and Cancer-associated fibroblasts (CAFs) (Fig. 5d and Supplementary Fig. S7b and S8a). Although all 3 subsets were distributed across most patients, we did not identify any CAFs or stellate cells for 2 of them. Similarly, we performed sub-clustering for B cells, T cells and macrophages (Supplementary Fig. S8a, b).

### Poor prognosis patients demonstrate an upregulation in crosstalk between macrophages, stromal cells, and cancer cells

Based on ligand-receptor expression, we assessed the cellular crosstalk between different cell populations. To do so, we applied the CrossTalkeR tool to directly compare changes in cell-cell communication between the two prognosis groups^34^. For this we focused mainly on subsets showing the largest differences in cell-cell interactions between the prognosis groups. This included Macrophages, Stellate cells, Endothelial cells EMT-like cancer cells and Fibroblast-like cancer cells. We observed a shift in cellular communication towards Fibroblast-like cancer cells in the poor prognosis group. This is mainly driven by communication with macrophages, CAFs and EMT-like cancer cells (Fig. 5e and Supplementary Fig. S9a). The top ligand in the crosstalk from cancer cell cluster 4 to the stromal and macrophage myeloid cell clusters was the Versican gene, VCAN. VCAN encodes an extracellular matrix protein known to be involved in cancer progression and inflammation^35^. VCAN was predicted to interact with Toll-like receptor 2 (TLR2) on macrophages (Fig. 5f) and epithelial growth factor receptor (EGFR) in stromal cells (Supplementary Fig. S9a). Among the top ligands driving communication from the macrophage myeloid cell cluster was the tumor growth factor β (TGF-β) gene (Supplementary Fig. S9b), TGFB1. TGF-β has two receptors, type I and type II, and is a well-known regulator of cancer immune evasion as well as other aspects of tumor progression^36^. We also found AXL among the ligand-receptor pairs mainly present in the poor prognosis patients. AXL is a member of the three known TAM receptors (Tyro3, MerTK, AXL) interacting with its ligands GAS6 and PROS1. Specifically, GAS6 – AXL interaction was predicted between GAS6-expressing stellate cells CAFs and macrophages and AXL-expressing macrophages, CAFs Fibroblast like cancer cells (Fig. 5f). TAM receptors (TAMr) are well-known regulators of macrophage, cancer and stromal cell function and are potent inhibitors of the inflammatory response.

### Poor prognosis signature is in a large subset of eCCA patients in available transcriptomic data

**Fig. 6:**
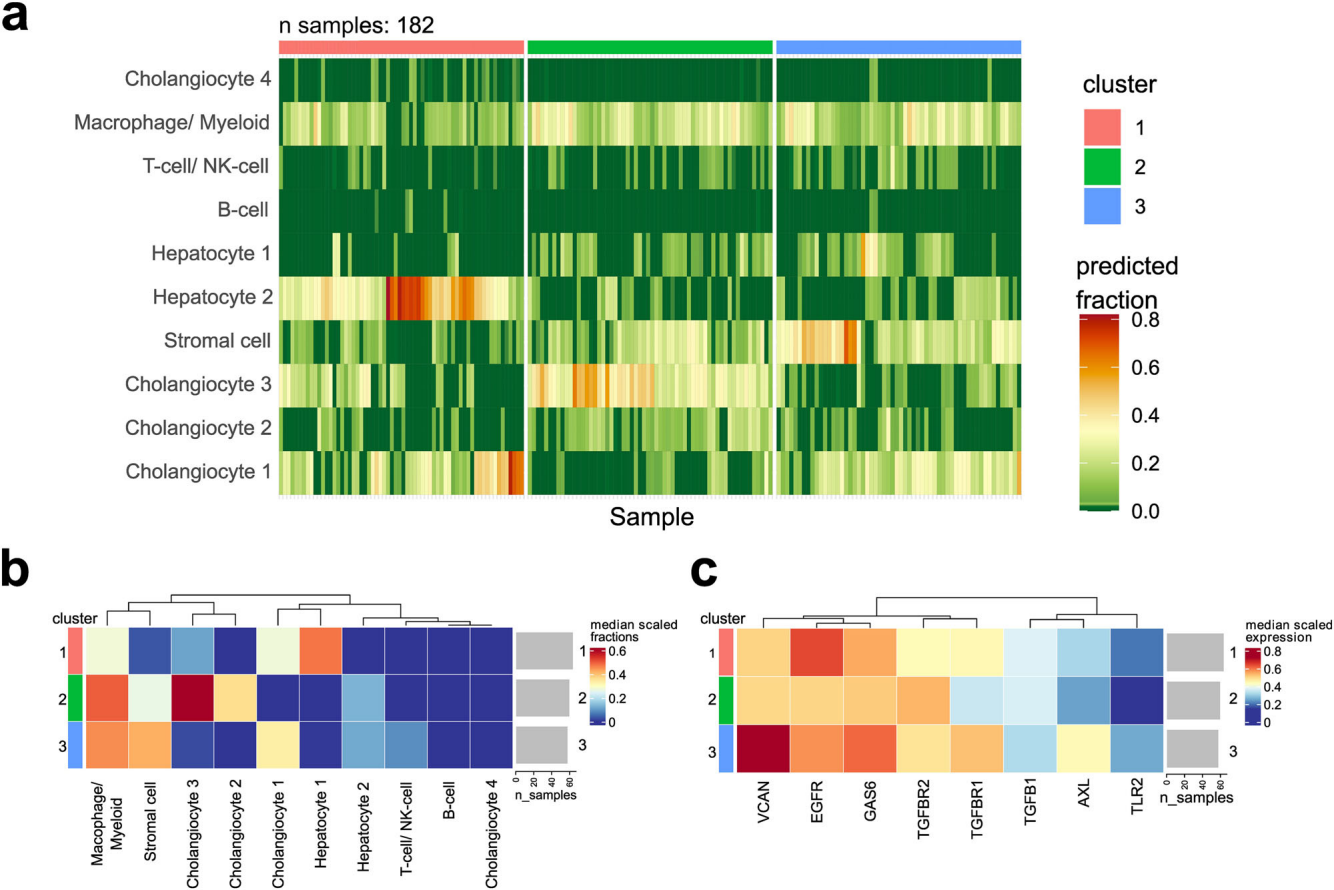
Poor prognosis signature is common in a subset of eCCA patients. Deconvolution of transcriptional data of 184 eCCA samples based on cibersortx using signatures generated from the clusters in our snRNA data. **a** Relative fraction predicted by cibersort for each sample. Samples were clustered into 3 distinct clusters based on the relative fractions. **b** Median relative fractions for the identified clusters. Cluster 3 is enriched in stromal cells and has a high abundance of macrophage/myeloid cells. **c** Gene expression of genes, identified to be involved in the cellular crosstalk in the TME of patients with a poor prognosis. Cluster 3 has high expression of VCAN-EGFR and GAS6-AXL ligand-receptor pairs.

To validate the transcriptomic findings in our pCCA cohort, we analyzed publicly available microarray data from 182 cases of extrahepatic CCA (eCCA). The dataset contains dCCA cases as well, but the majority of 130 cases consist of pCCA^37^. To assess cell type abundance, we performed cellular deconvolution using CIBERSORTx^38^ based on signatures generated from the 10 clusters identified in our snRNA seq data. These predicted fractions were used to cluster the patients into 3 groups (Supplementary Fig. S10a, b). While cancer cell clusters 1-3, stromal cells, both hepatocyte clusters and the macrophage/myeloid cell cluster were predicted to be abundant, cancer cell cluster 4, B-cell and T-cell/NK-cell fractions were predicted to be low (Fig. 6a). Cluster 3 including 58 patients (32%) was predicted to be particularly rich in macrophages and stromal cells (Fig. 6b). The same cluster expresses high levels of VCAN, EGFR, GAS6, and AXL (Fig. 6c) suggesting the involvement of the two ligand-receptor pairs we identified in the poor prognosis patients in our snRNA sequencing data.

### Ligand-receptor pairs are expressed in close proximity

**Fig. 7:**
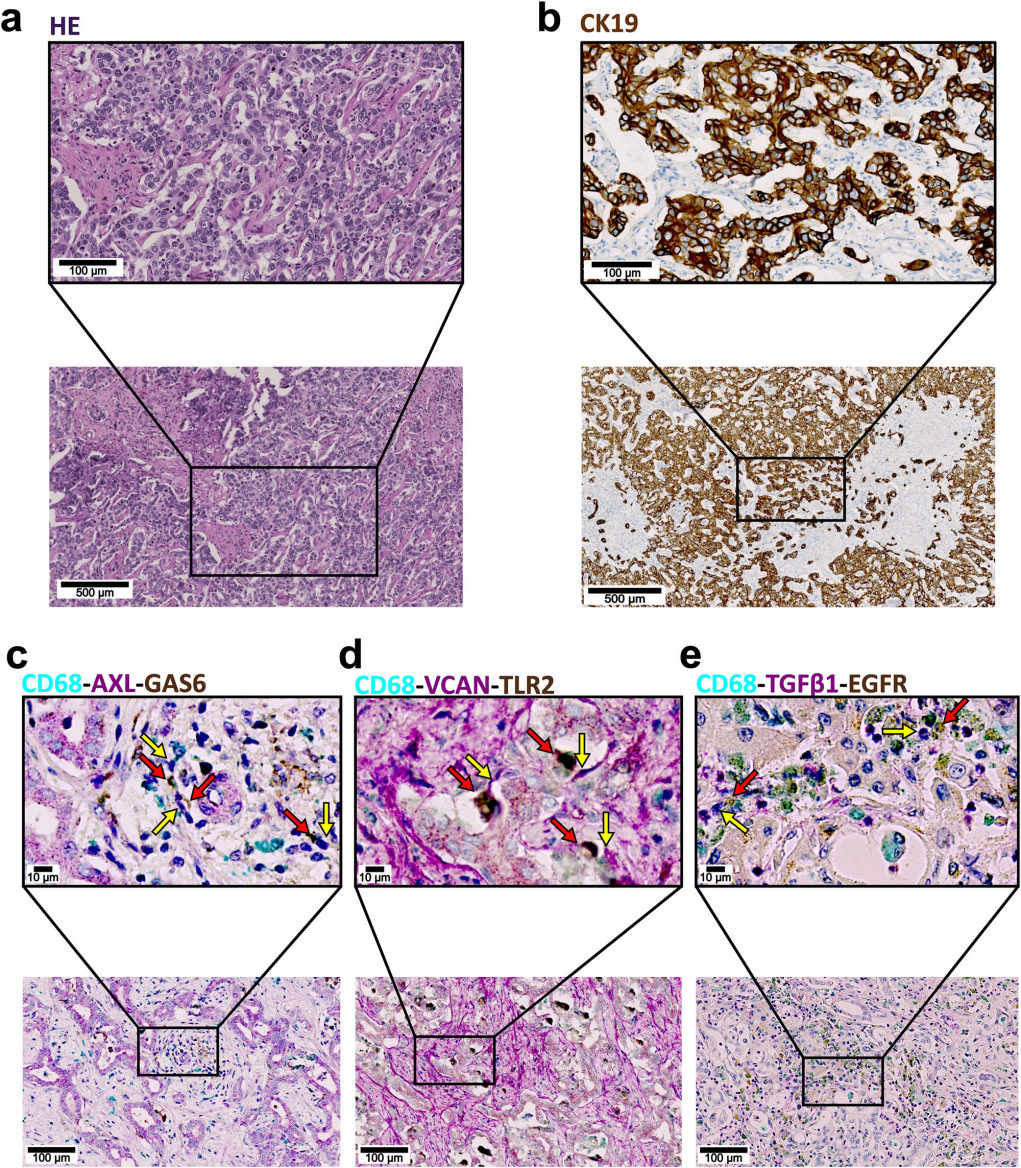
Validation of Ligand-Receptor interactions. Direct interaction of Ligand-receptor pairs identified in snRNA was assessed using Co-Immunohistochemistry (IHC). **a** HE-staining and **b** staining of Cytokeratin 19 (CK19) in representative CCA tumor regions. **c** Staining of Macrophage marker CD68 and the Ligand receptor pair GAS6-AXL. Co-expression of CD68 with AXL receptor can be seen by overlapping stainings (yellow arrow). GAS6-expressing cells (red arrow) are present in the proximity of the AXL-expressing macrophages. **d** Extracellular matrix protein VCAN expression in the extracellular space (yellow arrows) is in direct contact with TLR2-expressing CD68 positive macrophages (red arrow). **e** Staining of TGFβ1 (yellow arrow) in proximity to EGFR expressing cells (red arrow).

To confirm the expression of the predicted ligand-receptor pairs on a protein level, we used multiplexed immunohistochemistry. This also allowed us to examine whether they are expressed in proximity. We confirmed the presence of cancer based on HE stains and Cytokeratin 19 (CK19) stainings (Fig. 7a and b). We were able to confirm the expression of AXL in CD68 positive macrophages near GAS6 expressing cells (Fig. 7c). We observed abundant VCAN in the extracellular space. VCAN expression bordered TLR2 expressing macrophages (Fig. 7d). Similarly, we observe TGFβ1 expressing macrophages in proximity to EGFR expressing cells (Fig. 7e).

## Discussion

CCA treatment options remain limited, and even after decades of research, surgery still represents the best chance for a cure. While the role of immunotherapy is increasing in other cancer entities, knowledge about immune responses to cancer cells in CCA remains limited. Furthermore, studies are complicated by CCA heterogeneity, including a wide range of genetic subtypes^39^. The immune system can destroy cancer cells, but there is also a complicated immune activation network regulated by immune checkpoints^40^. The TME of CCA features a T-cell exclusion mechanism and is dominated by low cytotoxic immune cell infiltration and high levels of Tregs and checkpoint expression, which hinders the immune response against tumors^5^. Here, we report the contributions of different immune cells to the tumor microenvironment in iCCA and pCCA, with detailed definitions of their components.

Overall, the presence of many CD4 and CD8 cells in the tumor area is a sign of good prognosis, and previous studies have shown higher levels of CD4 and CD8 cells in the tumor margin than in the central tumor area in patients with iCCA^41,42^. Accumulation of Tregs in tumors correlates with poor prognosis^9,43^. Both iCCA and pCCA samples were infiltrated with Tregs and exhausted CD4 and CD8 tissue-resident memory cells, while CD8 effector memory cells were mostly excluded. Moreover, we identified elevated levels of CD69 in infiltrating Tregs. Patients with those activated Tregs, also demonstrated other subsets with multiple co-expression of checkpoints, indicating an exhausted phenotype. CD69, as an activation marker, is involved in Treg function and has been reported to be correlated with tumor progression in hepatocellular carcinoma^44,45^. PD-1 is a co-inhibitory receptor that can be expressed in many different cell types, including Tregs, activated B cells, and T cells^19,46^. Our data showed high levels of PD-1 as well as other checkpoint inhibitors such as TIGIT and CTLA-4, confirming a dominant exhausted phenotype. Interestingly, in 24% of our patients, we identified CD4 and CD8 residential memory cells with expression of PD-1, this indicates a group of patients who could potentially benefit from immune blockade therapy.

Our transcriptomic data revealed the TME in a broader perspective and identified the crosstalk between cell clusters responsible for the immunosuppressive environment. We identified several immune populations, as well as clusters of CAFs, cancer cells and a cluster of stellate cells, which are believed to be a major source of CAFs^9,47^. Macrophages were identified within the immune compartment and are known to contribute to immunosuppressive cell crosstalk within iCCA^48^. Following previous studies, we demonstrated increased expression of genes associated with a worse prognosis in the cancer cells, including MDM2 and PTPRM. MDM2 has been associated with advanced CCA stages and a worse clinical outcome^49,50^. PTPRM is associated with a worse outcome in cervical cancer^51^.

In our pCCA transcriptomic data, we identified several Ligand-receptor pairs and confirmed that the corresponding proteins are expressed in neighboring cells. This included GAS6, and AXL as unique ligand-receptor pairs associated with poor prognosis. *GAS6* codes for the growth arrest-specific 6 protein, which can be recognized by the AXL receptor belonging to the TAM subfamily. TAM receptors (TAMr) play a role in the immune system but are also associated with tumor progression^52^. Both GAS6 and AXL are associated with worse patient survival in lung cancer^53^, and their signaling is associated with the recruitment and polarization of suppressive macrophages in hepatocellular carcinoma^54^. In addition to its immunosuppressive role, AXL and TAM signaling are generally associated with PD-L1 expression^55,56^. Furthermore, we observed a shift in the crosstalk between different cell clusters. In our poor prognosis group, we identified increased crosstalk between a cancer cell subpopulation, macrophages, and stromal cells. Communication with macrophages was partly driven by the *VCAN* gene encoding for the extracellular matrix (ECM) protein versican and toll-like receptor 2 (TLR2). This interaction has been identified to promote an immunosuppressive environment^57^ and to stimulate metastasis in cancer^58^. In addition, versican is predicted to interact with the epithelial growth factor receptor (EGFR) in stromal cells. Versican signaling via EGFR in breast cancer has been shown to promote proliferation^59^. EGFR is commonly overexpressed in CCA, which can be associated with unfavorable patient outcomes^60^. High versican-levels were linked to resistance against immunotherapy in gastric cancer patients pointing towards it prognostic potential^61^. We also identified the TGF-β gene as a major ligand expressed in macrophages. TAM-derived TGF-β is known to promote tumor progression^62^ and drive the epithelial-mesenchymal transition in hepatocellular carcinomas^63^. TGF-β is also a key mediator in the induction of Tregs and can directly inhibit cytotoxic T cell function^26,64^, making it a potent promoter for the suppression of T cells. In our poor prognosis group, we identified several ligand-receptor pairs, that are important mediators in the immune exhaustion of CCA and explain the limited immune cell-mediated anti-tumor response in this cancer entity.

In summary, we used high-dimensional phenotyping, transcriptomics, and multiplexed immunohistochemistry to provide a deeper insight into the immune exhausted phenotype in CCA. The multiple mechanisms leading to the exclusion of relevant immune cells needed for an anti-cancer response and mechanisms leading to active immune suppression are part of complex cell-cell crosstalk. Our findings confirmed the presence of Tregs and the lack of effector memory cells in the tumor.

New findings are the spatiality of the effector memory cells being more present in the peripheral tissue, for some reason these immune cells fail to reach the tumor niche.

Transcriptomic data identified the presence of specific cancer cells, mainly only present in the poor prognosis group. Here, we identified an upregulated crosstalk of these cancer cells with the myeloid compartment and the stromal cells. This crosstalk creates an immunosuppressive TME, worsening patient outcomes. Amongst the responsible ligand pairs are GAS6-AXL belonging to the TAM family. We then identified VCAN-TLR2 to be present and influencing the ECM in a way to supports immune exhaustion. Last, EGFR-TGF-β is expressed in macrophages and this finding is important in Tregs induction and blocking cytotoxic T cell function. Using deconvolution of previously published transcriptomic data in eCCA, we identified the involved cell types as well as VCAN, EGFR, GAS6, and AXL expression to be common in a subgroup of patients. This represents the poor prognostic group of patients with a more aggressive biology of the disease.

The immunosuppressive environment in CCA is a complex problem that needs to be tackled in the hope of creating better perspectives and therapy options for patients with CCA. In this cancer entity, only a limited response to checkpoint blockade has been observed. This study contributes to the understanding of the immunosuppressive environment and provides signaling pathways as drivers. The identified crosstalk between macrophages and stromal cells demonstrates the complex interactions involving multiple cell types that shape immunosuppression in the TME within CCA. A deep understanding of this network of interactions is needed before patient stratification for therapy, and new targets can be identified.

Good biomarkers for predicting a response to immune blockade treatment are still missing. PD-L1 immunohistochemistry is currently used in daily diagnostic care, and its expression in macrophages, immune cells, and cancer cells has been previously reported. Our study demonstrates that in addition to the presence of PD-L1 blocking immune cells in the tumor, the presence of immune cells outside the tumor in the peripheral surrounding liver tissue might be of importance for patient prognosis. A multiplexed panel can be used on peripheral liver biopsies to define the presence of CD4 and CD8 effector memory cells to predict an activated immune phenotype. We hypothesize that further research should investigate immune cell composition and expression of costimulatory and coinhibitory checkpoints in the tumor as well as the surrounding liver tissue. This might reveal response biomarkers for immunotherapy treatment. Although this is a relatively complex workup, multiplexed imaging is available in most university hospitals and may represent a step towards personalized cancer care in a patient group with sparse therapeutical options.

## Methods

### Patient recruitment

Samples were collected between May 2020 and June 2021 from 16 patients with primary iCCA (*n* = 7) or pCCA (*n* = 9) with a curative intent for surgery, all patients were treatment-naïve. One iCCA patient was excluded from the CyTOF cohort, due to low cell counts of the suspension of the paired peripheral liver sample; 6 iCCA and 9 pCCA patients were included in the final analysis. The presence of distant metastases was ruled out. Patient characteristics are shown in Table 1. Data has been collected in accordance with the Declaration of Helsinki, and all patients consented to participate in this study with local ethical approval under EK106/18 and EK360/19 and the broad consent (EK206/09) of the hospitals centralized biomaterial bank (RWTH cBMB).

### Sample collection and tissue digestion

Blood and tissue samples were collected from each patient. Blood was collected before the operation in 20 ml EDTA tubes. Blood samples were processed within 4 h of blood withdrawal. PBMCs were isolated using Ficoll after the addition of 20 mL of 2% FBS/PBS. After centrifuging for 20 min at 1300 *g* without break, we removed the PBMC ring using a pipette. This was then washed and centrifuged again at 300 *g* and if red blood cells were lysed with ACK lysing buffer the cells were washed again.

Tissue samples were collected postoperatively from the pathology department of our hospital. Tissue blocks of normal tissue and tumor tissue with a size of 10 × 10 × 5 mm were placed in RPMI medium (Gibco), which was cooled on ice. Our digestion protocol was used as in our previous study^65^. The tissue was minced with a scalpel, and enzymes were added for digestion. This solution, along with the tissue pieces, was placed in a shaker for 40 min at 37 degrees Celsius (°C). Then, the medium was refreshed, new enzymes were added and again placed into the shaker for an additional 30 min. The remaining solid tissue components were pushed through using a cell strainer, providing the greatest possible yield. The tube was centrifuged at 300 *g* for 10 min, and the pellet was collected and cryopreserved in a medium containing DMSO at −80 °C until further analysis.

### CyTOF sample preparation

The metal-isotope labeled antibodies used in this study were conjugated using the MaxPar X8 Antibody labeling kit according to the manufacturer’s instructions (Fluidigm) or were purchased from Fluidigm pre-conjugated. Each conjugated antibody was quality-checked and titrated to the optimal staining concentration using a combination of primary human cells (peripheral blood mononuclear cells, single-cell suspensions from tonsils, and single-cell suspension from cholangiocarcinoma). The samples were thawed for further CyTOF staining by gently diluting DMSO in warm RPMI and washing 2 times in RPMI. Single-cell suspensions of cholangiocarcinoma tumor tissue, surrounding normal liver tissue, and peripheral blood mononuclear cells were thawed and washed with warm culture media (RPMI + 10%FCS Pen/strep/glut). The cells were incubated with culture medium + 15 U/ml DNase for 10 min at 37 °C. Cells were washed in PBS and incubated with 0.5 μM cisplatin (Fluidigm) in phosphate-buffered saline (PBS) for 5 min at room temperature (RT) to label non-viable cells. The cells were resuspended in Truestain FC blocker (BioLegend) for 10 min at RT. After incubation, an antibody mix of targets not capable of fixation (CCR5, CXCR3, CCR, and IL-7RA) was added to the cells for 30 min at 4 °C. The cells were fixed with 1.6% PFA in PBS for 10 min at RT. Cells were washed with CSM and fixed with 1.6% PFA in PBS for 10 min at room temperature (RT). Fixed cells were palladium barcoded (Fluidigm) according to established protocols. All surface staining was performed in CSM for 30 min at RT (antibodies listed in Supplementary Table 2). Cells were washed in CSM and permeabilized using the FoxP3 staining kit, according to the manufacturer’s instructions (eBioscience™ FoxP3/Transcription Factor Staining Buffer Set, Invitrogen. After intracellular staining, cells were fixed with 1.6% PFA in PBS for 10 min at RT. Before acquisition, samples were washed in CSM and resuspended in intercalation solution (1.6% PFA in PBS, 0.02% saponin (Sigma), and 0.5 μM iridium diluted in Fix/Perm solution (Fluidigm)) for 1 h at RT or overnight at 4 °C or frozen in aliquots to label DNA. Before acquisition, the cells were washed once with RPMI + 30% FCS. The samples were washed twice with CSB and once with larger volumes of CAS. All samples were filtered through a 35 μM nylon mesh cell strainer, resuspended at 0.5 × 10^6^ cells/mL in CAS supplemented with 1/10 EQ four-element calibration beads (Fluidigm), and acquired on a CyTOF2 mass cytometer (Fluidigm) using the Super Sampler injection system. Before data acquisition, the instrument was demonstrated to have Tb159 dual counts greater than 1,000,000 and oxidation less than 3%; if the instrument failed these criteria, it was cleaned, tuned, or repaired as necessary.

### CyTOF data acquisition and pre-analysis

Immediately before the acquisition, samples were washed as described above and then resuspended at a concentration of 0.5 million cells/mL in cell acquisition solution containing a 1/10 dilution of EQ 4 Element Beads (Fluidigm). Samples were acquired on a CyTOF Helios mass cytometer at an event rate of <150 events/s. After the acquisition, the data were normalized using bead-based normalization in CyTOF software. The data were exported as FCS files for downstream analyses. The data were gated to exclude residual normalization beads, debris, dead cells, and doublets, leaving DNA+ live single events for subsequent clustering and high-dimensional analysis. The total number of viable CD45+ cells acquired per sample is listed in the supplement (Supplementary Table 4).

### Batch effect correction

Data were acquired in five different batches, each containing two technical replicates, one single-cell suspension of tonsil cells, and one PBMC sample. We used CytoNorm^66^ to model the differences in signal intensities between batches. We determined which of the two replicate sets had a larger dynamic range per marker. TIGIT, CD69, CTLA-4, ICOS, PD-1, CXCR3, LAG-3, OX40, CD7, CD95, CD45RA, CD45, CD127, and HLA-DR were modeled and normalized using tonsil replicates. All other markers were modeled using PBMC replicates. The data were arcsinh-transformed (factor 5) and clustered into B cells, T cells, NK cells, and myeloid lineages using FlowSOM. Subsequent settings for CytoNorm were as follows: 21 quantiles from the 0th to 99th percentile. The limits were (0 and 7.4). All FCS files were normalized using both normalization models.

### CyTOF data analysis

After batch correction was performed, the data were further analyzed using R (v. 4.2.2). Subsequently, cells were clustered using the FlowSOM^67^ algorithm (CATLYST package v. 1.22.0) based on CD2, CD3, CD4, CD8, CD14, CD45, and HLA-DR on a 10 by 10 grid and with elbow metaclustering. The algorithm resulted in eight metaclusters that could be manually assigned to CD4 T cells (FlowSOM metaclusters 1 and 4), CD8 T cells (FlowSOM metaclusters 2 and 3), and miscellaneous cells, included NK cells, CD14+ cells, and others (FlowSOM metaclusters 5-8).

Cells were downsampled to less than 50000 CD4 and CD8 cells per sample, resulting in 918.019 CD4 T cells and 732.047 CD8 T cells. OptSNE dimensionality reduction from the MulticoreTSNE package (v.0.1) using Python (v.2.7.18) with a perplexity of 30, theta of 0.5, maximum of 1000 iterations, and stop of 5000 was performed using CD45RA, CD45RO, CCR7, CD27, CD28, CD69, CD103, CD95, CD57, CD161, CD44, CD25, CD127, FOXP3, CXCR3, CCR5, CCR4, CD11a, and CD49d. Subsequently, Phenograph^68^ (Rphenograph package v. 0.99.1) was applied using the same markers with K-nearest neighbors set to 20 and Euclidean as the distance metric. The resulting clusters (25 for CD4 + T cells and 31 for CD8 + T cells) were annotated according to their expression profiles into known immune cell subset categories for data interpretation. Markers used to define different subsets are mentioned in the supplement (Supplementary Table 2).

Statistical comparisons involved Friedman’s test to compare mean frequencies and marker expression across tissues in either iCCA or pCCA (matched blood, normal tissue, and tumor tissue samples), Mann-Whitney test to compare mean frequencies and marker expression between iCCA and pCCA tumors, and Wilcoxon test to compare mean frequencies and marker expression between normal and tumor tissues. Correlation analysis between cluster frequencies in blood versus tumor tissues and mean marker expression was performed using Spearman’s test.

### Histology and multiplexed immunohistochemistry of Immune markers

The histology report of each patient was checked for quality control of the right tumor. The pathologist (LH) collected the H&E-stained slides and selected the tumor block. Sections were cut using a Leica RM2235 microscope at a thickness of approximately 4 μm. Antibodies against CK19, CD4, CD8, CD103, and PD-1 were analyzed, and their co-expression was quantified using the TissueFAXS PLUS scanning system with StrataQuest Analysis Software from TissueGnostics^69^, Vienna, Austria.

#### Single-nuclei isolation

In addition to cytometric analysis, we also chose seven patients for transcriptomic analysis using single nuclei RNA sequencing (snRNA seq). The patients were divided into two groups based on their clinical prognosis. For the four patients in the poor clinical prognosis, we detected lymph node invasion by the tumor and recurrence within 1 year of surgery. In the three patients in the good prognosis group, no lymph node invasion was detected, and they were recurrence-free at the time of the study (at least one year after surgery). NGS data was available in 3 patients, see Supplementary Table 3 Tumor samples were frozen in cryotubes without fluid as quickly as possible after sample collection. To do so, they were covered in liquid nitrogen and then transferred to a − 80 °C freezer for long-term storage. Single-nuclei isolation was performed as described previously^70^.

Briefly, tissue samples were cut into pieces <0.5 cm and homogenized using a glass Dounce tissue grinder (Sigma-Aldrich). The tissue was homogenized by subjecting it to 25 strokes with pestle A and 25 strokes with pestle B in 2 ml of ice-cold nuclei EZ lysis buffer. Subsequently, the cells were incubated with an additional 3 ml of cold EZ lysis buffer on ice for 5 min. Nuclei were centrifuged at 500 *g* for 5 min at 4 °C, washed with 5 ml ice-cold EZ lysis buffer, and incubated on ice for 5 min. After centrifugation, the resulting nuclei pellet was washed with 5 ml nuclei suspension buffer (NSB; consisting of 1× PBS, 0.01% BSA, and 0.1% RNase inhibitor). Isolated nuclei were resuspended in 2 ml NSB, filtered through a 35 μm cell strainer, and counted. Approximately 1000 nuclei per µL were used for loading on a 10x channel.

#### snRNA-seq preprocessing, clustering, and annotation

From the single nuclei using 10X sequencing, we generated raw reads that were further processed by Cellranger version 6.1.2, using GRCh38-2020-A as the human reference transcriptome. This gave us a count matrix that was converted to a fast q file by the default mkfastq function, followed by alignment using the count function, cell calling, and quantification of UMI counts. Only cells that expressed between 1000 and 20,000 genes were retained using the filter_cells function. Furthermore, cells with more than 10% of the reads mapped to the mitochondrial genes were removed. To exclude potential doublets, the Scrublet tool was used, with a cutoff of 0.2. For subsequent analysis, we used Seurat (v. 4.3.0). The expression data were log-normalized with a scaling factor of 10,000. We applied the Seurat V3 method to select the top 2000 highly variable genes that were subsequently used to calculate the principal components of the data. The harmony package was utilized as a batch-effect correction tool and set on sample IDs as the batch variable. UMAP was applied with Seurat’s RunUMAP function using the first 30 principal components generated from the harmony corrected PCA embedding. The cells were clustered using the Louvain algorithm at resolutions ranging from 0.3 to 1.1. To determine the final resolution, we manually inspected the marker genes and cluster composition. The dataset used for further downstream applications consisted of 20,995 cells from 7 human CCA samples. Clusters were annotated using curated marker genes from literature.

#### Differential expression analyses

We used the MAST algorithm to identify differentially expressed genes in all cell types based on the prognosis groups. First, genes with low expression and normalized expression below zero were removed. The hurdle model was set with the good prognosis group as the reference condition and the poor prognosis as our condition. We extracted the differentially expressed genes, with the logarithm of fold change (logFC) value and adjusted p values from the summary statistics of the output. The Benjamini-Hochberg FDR correction method was used to identify statistically significant DE genes. Gene set enrichment analysis (GSEA) of differentially expressed genes was performed using the clusterProfiler package (v. 3.17) with a minimal gene set size of 3, a maximal gene set size of 500, and a p-value cutoff of 0.05 using Gene Ontology annotations.

#### Cell-cell communication analyses

To predict ligand-receptor (LR) interactions between the previously identified clusters, we used the CrossTalkeR package (v. 2.0.0). The *liana_wrap* function of liana (v. 0.1.14) was used to generate LR prediction. As an input, CrossTalkeR uses cellphoneDB predictions as a database and generates a comparative cell-cell interaction (CCI) network for the conditions. We performed the CCI analysis for main clusters, sub-cluster and selected sub-clusters of interest, comparing poor prognosis with respect to good prognosis. Further, we looked for a few handpicked ligands and receptors to show their interaction in different sub-cluster groups.

### Analysis of public expression data

Transcriptome array data were retrieved from the Gene Expression Omnibus (GEO) dataset GSE132305 for 182 patients with eCCA, in this cohort 130 cases of pCCA were included and processed according to the authors’ original methodology^37^. For cellular deconvolution, expression data were exported and analyzed using the CIBERSTORx tool^38^. The signature matrix file was generated using the snRNA and clusters from this study as a single cell reference, as described in the original article. For the signature matrix, we used the top 50 DE genes from each of the 10 clusters resulting in 337 unique genes. The estimated relative fractions were used as input for clustering via phenograph.

### Multiplexed immunohistochemistry of Ligand-receptor pairs

Co-immunohistochemistry staining were performed in 4 µm-thick paraformaldehyde-fixed paraffin-embedded liver tissue sections using the Discovery Ultra Automated Slide Preparation System (Roche), as previously described^71,72^. Briefly, after deparaffinized and antigen retrieval steps, the slides were incubated with the first primary antibody (Supplementary Table 5). Appropriate secondary antibodies were automatically added by the system, followed by incubation with the teal kit (Supplementary Table 5). The slides were then heated to 95 °C to denature the first primary antibody. The second primary antibody was then added manually, followed by automatic incubation with an appropriate secondary antibody and the DAB kit. After denaturation steps, the third primary antibody was added manually, followed by automatic application of an appropriate secondary antibody and incubation with the purple kit (Supplementary Table 5). The slides were then counterstained with hematoxylin II and bluing reagent. Whole slide scans were acquired using the Axio Scan.Z1 (Zeiss), and representative snapshots are shown in the results section.

## Supplementary information

Supplementary information

## Data Availability

The code used in analyzing this data is available at https://github.com/hayatlab/cholangiocarcinoma_ici. The raw data generated in this study using single nuclei RNA-sequencing will be made available via the European Nucleotide Archive (ENA) under the accession code PRJEB97203. Bulk transcriptomic data was retrieved from the Gene Expression Omnibus (GEO) dataset GSE132305.
