# Supplementary material for: Multimodal single-cell profiling reveals crosstalk between macrophages and stromal cells in poor prognostic cholangiocarcinoma patients
Source: NPJ Precis Oncol. 2026 Jan 28;10:105. doi: 10.1038/s41698-026-01292-6 (PMC12976350; doi:10.1038/s41698-026-01292-6)
Supplement: Supplementary file 1 — Supplementary information [file 41698_2026_1292_MOESM1_ESM.docx]

**Supplementary Items**

**Multimodal single-cell profiling reveals crosstalk between macrophages and stromal cells in poor prognostic cholangiocarcinoma patients**

Lara Heij^1,2,3^*, Sikander Hayat^3^*, Konrad Reichel^1,4,5^*, Sidrah Maryam^3^, Colm J. O’Rourke^6^, Xiuxiang Tan^7^, Marlous van den Braber^4,8^, Jan Verhoeff^4,8,9^, Maurice Halder^3^, Fabian Peisker^3^, Georg Wiltberger^7,10^, Jan Bednarsch^1^, Daniel Heise^1^, Julia Campello Deierl^7^, Sven A. Lang^1^, Florian Ulmer^1^, Tom Luedde^11^, Edgar Dahl^12,13^, Danny Jonigk ^12,13,14,15^, Jochen Nolting^1^, Shivan Sivakumar^16^, Jens Siveke^17^, Florian Vondran^7^, Flavio G. Rocha^18^, Hideo A. Baba^2^, Sylvia Hartmann^2^, Jesper B. Andersen^6^, Zaynab Hobloss^19^, Ahmed Ghallab^19,20^, Jan G. Hengstler^19^, Juan J. Garcia Vallejo^4,8§^ , Rafael Kramann^3§^, Ulf Neumann^1,7,10§^

Table 1. antibodies used for CyTOF

| Target protein | Clone | Metal | Manufacturer | Catalogue number | Dilution |
| --- | --- | --- | --- | --- | --- |
| Barcodes |  | 103-110Pd | Standard BioTools | 201060 |  |
| Iridium |  | 191-193Ir | Standard BioTools | 201192B | 1:1000 |
| Cisplatin |  | 194-195Pt | Standard BioTools | 201196 | 1:1000 |
| CD45 | HI30 | 89Y | Standard BioTools | 3089003B | 1:83 |
| CD49d | 9F10 | 141Pr | Standard BioTools | 3141004B | 1:50 |
| CD11a | HI111 | 142Nd | Standard BioTools | 3142006B | 1:83 |
| CD5 | UCHT2 | 143Nd | Standard BioTools | 3143007B | 1:83 |
| CD195 (CCR5) | NP-6G4 | 144Nd | Standard BioTools | 3144007A | 1:100 |
| CD4 | RPA-T4 | 145Nd | Standard BioTools | 3145001B | 1:50 |
| CD8a | RPA-T8 | 146Nd | Standard BioTools | 3146001B | 1:50 |
| CD7 | CD7-6B7 | 147Sm | Standard BioTools | 3147006B | 1:83 |
| CD103 | Ber-ACT3 | 148Nd | BioLegend | 130-122-303 | 1:100 |
| CD25 (IL-2R) | 2A3 | 149Sm | Standard BioTools | 3149010B | 1:50 |
| CD134 (OX40) | ACT35 | 150Nd | Standard BioTools | 3150023B | 1:50 |
| CD2 | TS1/8 | 151Eu | Standard BioTools | 3151003B | 1:83 |
| CD95 (Fas) | DX2 | 152Sm | Standard BioTools | 3152017B | 1:50 |
| CD366 (TIM-3) | F382E2 | 153Eu | BioLegend | 345019 | 1:100 |
| CD14 | 61D3 | 154Sm | Hybridoma |  | 1:200 |
| CD279 (PD-1) | EH12.2H7 | 155Gd | Standard BioTools | 3155009B | 1:50 |
| CD183 (CXCR3) | G025H7 | 156Gd | Standard BioTools | 3156004B | 1:100 |
| CD194 (CCR4) | L291H4 | 158Gd | Standard BioTools | 3158032A | 1:100 |
| CD197 (CCR7) | G043H7 | 159Tb | Standard BioTools | 3159003A | 1:50 |
| CD28 | CD28.2 | 160Gd | Standard BioTools | 3160003B | 1:50 |
| CD152 (CTLA-4) | 14D3 | 161Dy | Standard BioTools | 3161004B | 1:50 |
| CD69 | FN50 | 162Dy | Standard BioTools | 3162001B | 1:50 |
| TIGIT | MBSA43 | 163Dy | ThermoFisher | 16-9500-82 | 1:100 |
| CD161 | HP3G10 | 164Dy | Standard BioTools | 3164009B | 1:50 |
| CD45RO | UCHL1 | 165Ho | Standard BioTools | 3165011B | 1:50 |
| CD44 | BJ18 | 166Er | Standard BioTools | 3166001B | 1:70 |
| CD27 | 323 | 167Er | Standard BioTools | 3167002B | 1:50 |
| CD278 (ICOS) | C398.4A | 168Er | Standard BioTools | 3168024B | 1:40 |
| CD45RA | HI100 | 169Tm | Standard BioTools | 3169008B | 1:70 |
| CD3 | UCHT1 | 170Er | Standard BioTools | 3170001B | 1:83 |
| GITR | 621 | 171Yb | BioLegend | 311602 | 1:100 |
| CD57 | HCD57 | 172Yb | Standard BioTools | 3172009B | 1:67 |
| FoxP3 | 206D & 259D | 173Yb | BioLegend | 320102 and 320202 | 1:50 |
| HLA-DR | L243 | 174Yb | Standard BioTools | 3174001B | 1:67 |
| CD223 (LAG-3) | 11C3C65 | 175Lu | Standard BioTools | 3175033B | 1:50 |
| CD127 (IL-7Ra) | A019D5 | 176Yb | Standard BioTools | 3176004B | 1:100 |
| CD16 | 3G8 | 209Bi | Standard BioTools | 3209002B | 1:50 |


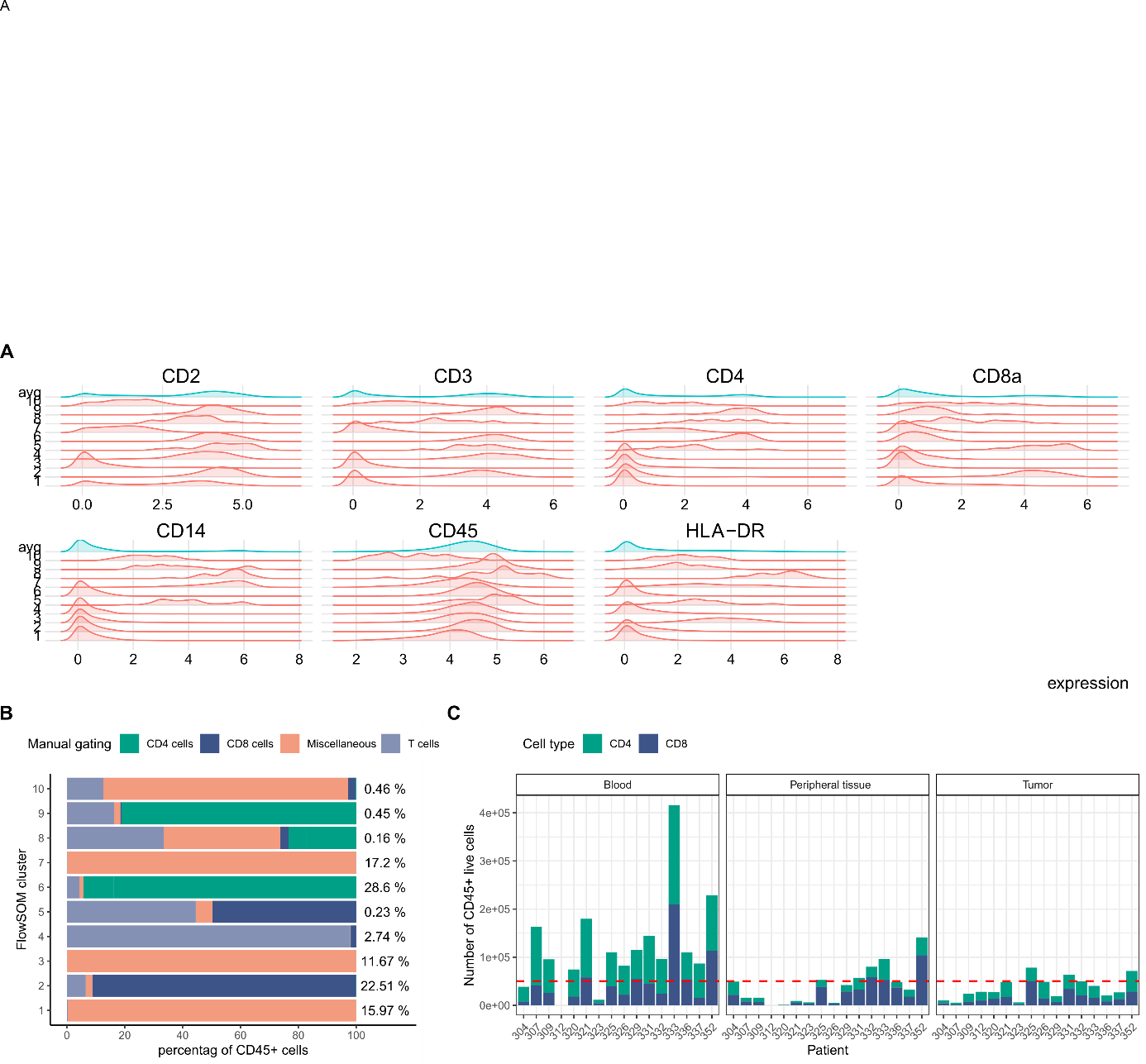


Figure S1. Gating and initial FlowSOM clustering

**(A)** initial clustering for identification of T cells. FlowSOM clustering based on shown lineage markers resulted in 10 clusters. Shown are density plots of normalized marker expression across the clusters **(B)** Percentage of cells shared between manual gating and the 10 clusters. Cluster 2 was identified as CD8 cells and cluster 6 was identified as CD4 cells. On the right, the percentage of CD45+ live cells in the given cluster is indicated **(C)** Total number of CD4 and CD8 cells in patients’ samples across tissues. Downsampling was performed on 50,000 cells or less (indicated with a red line).


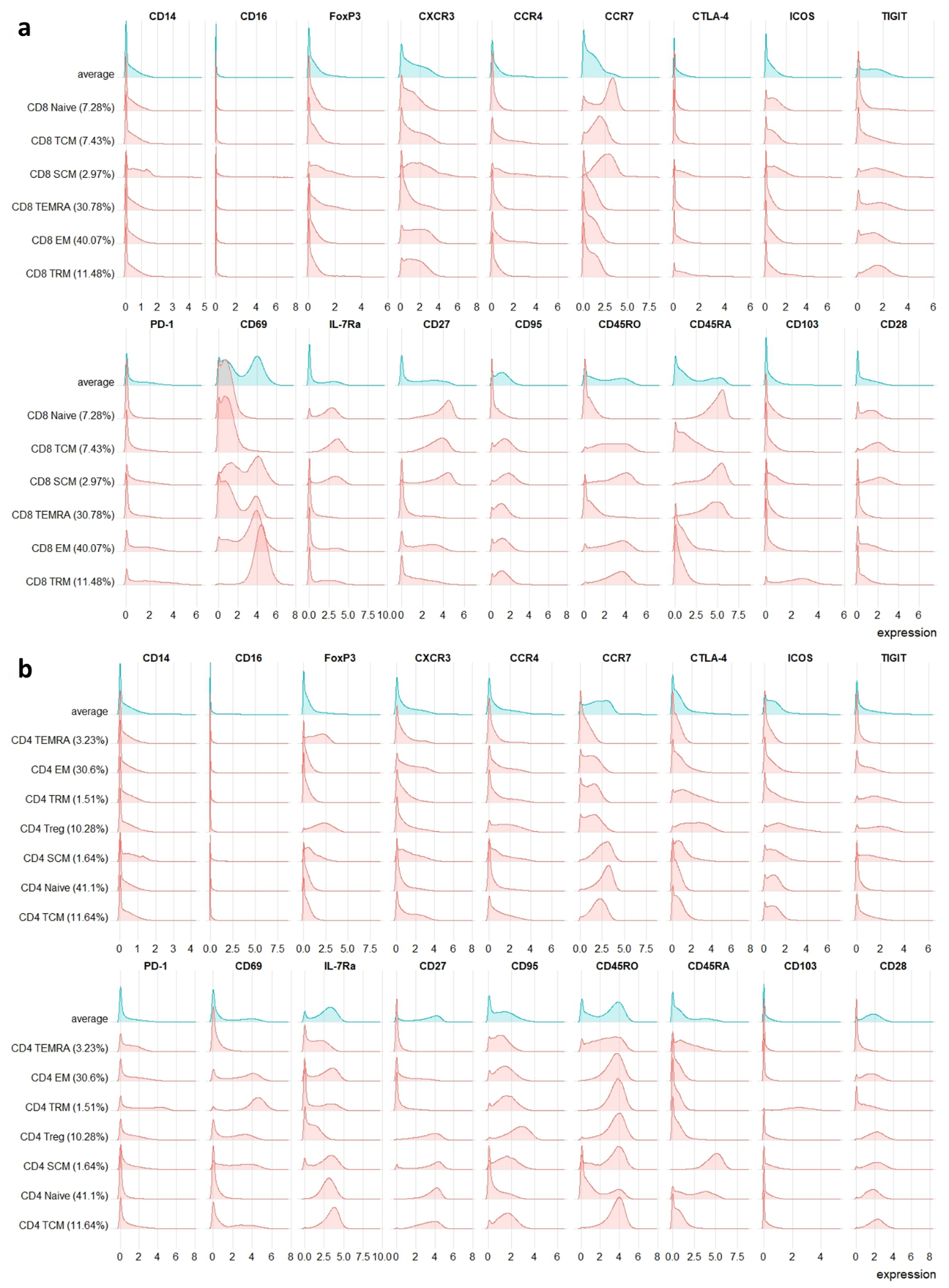


Figure S2. Marker expression in annotated cell types

Density plot of normalized marker expression. Cells were clustered and clusters were annotated based on the marker expression for **(a)** the CD8 dataset and **(b)** the CD4 dataset. The upper blue column (avg) shows the average expression of all CD4+/ CD8+ T cells.


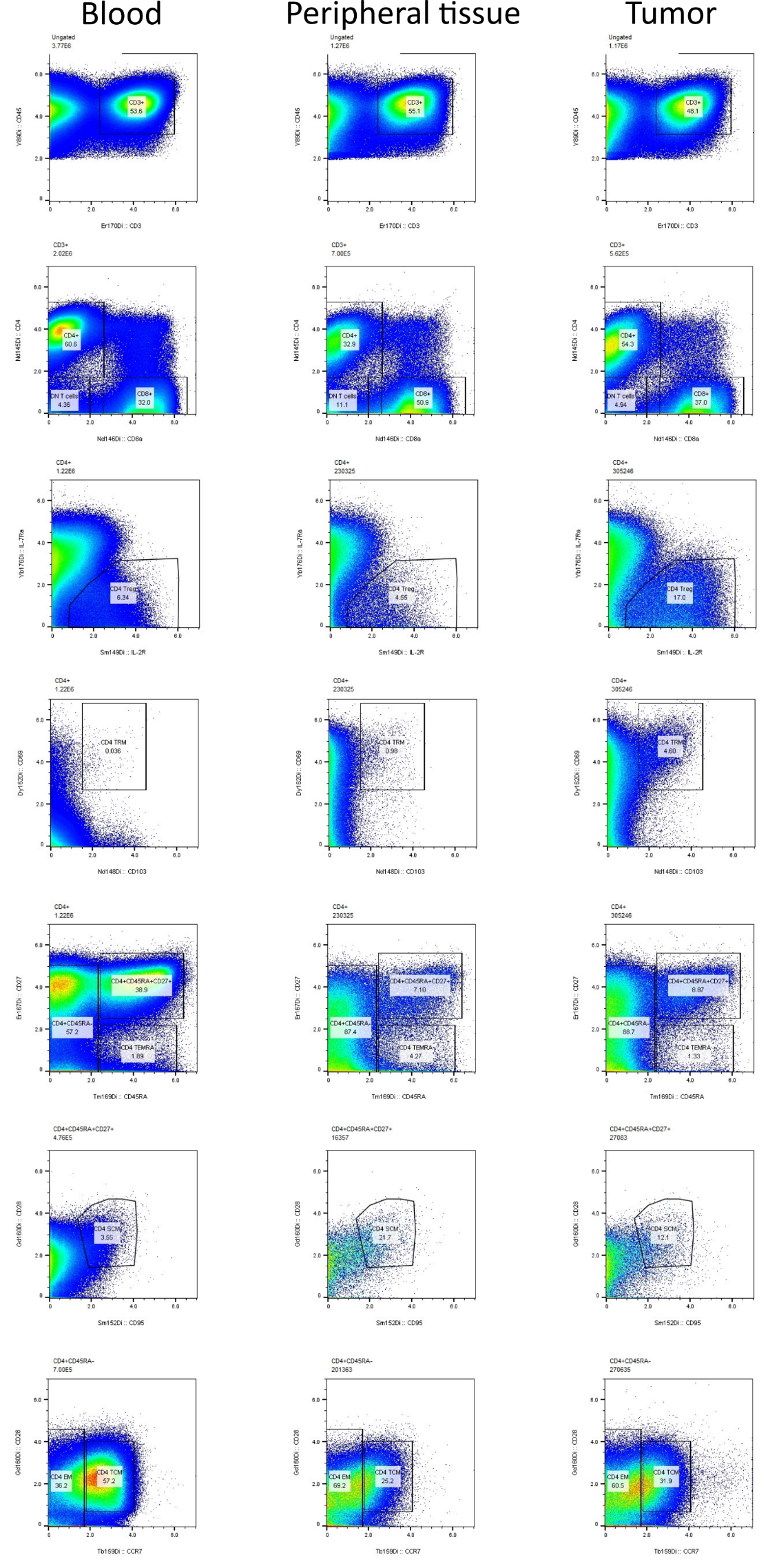


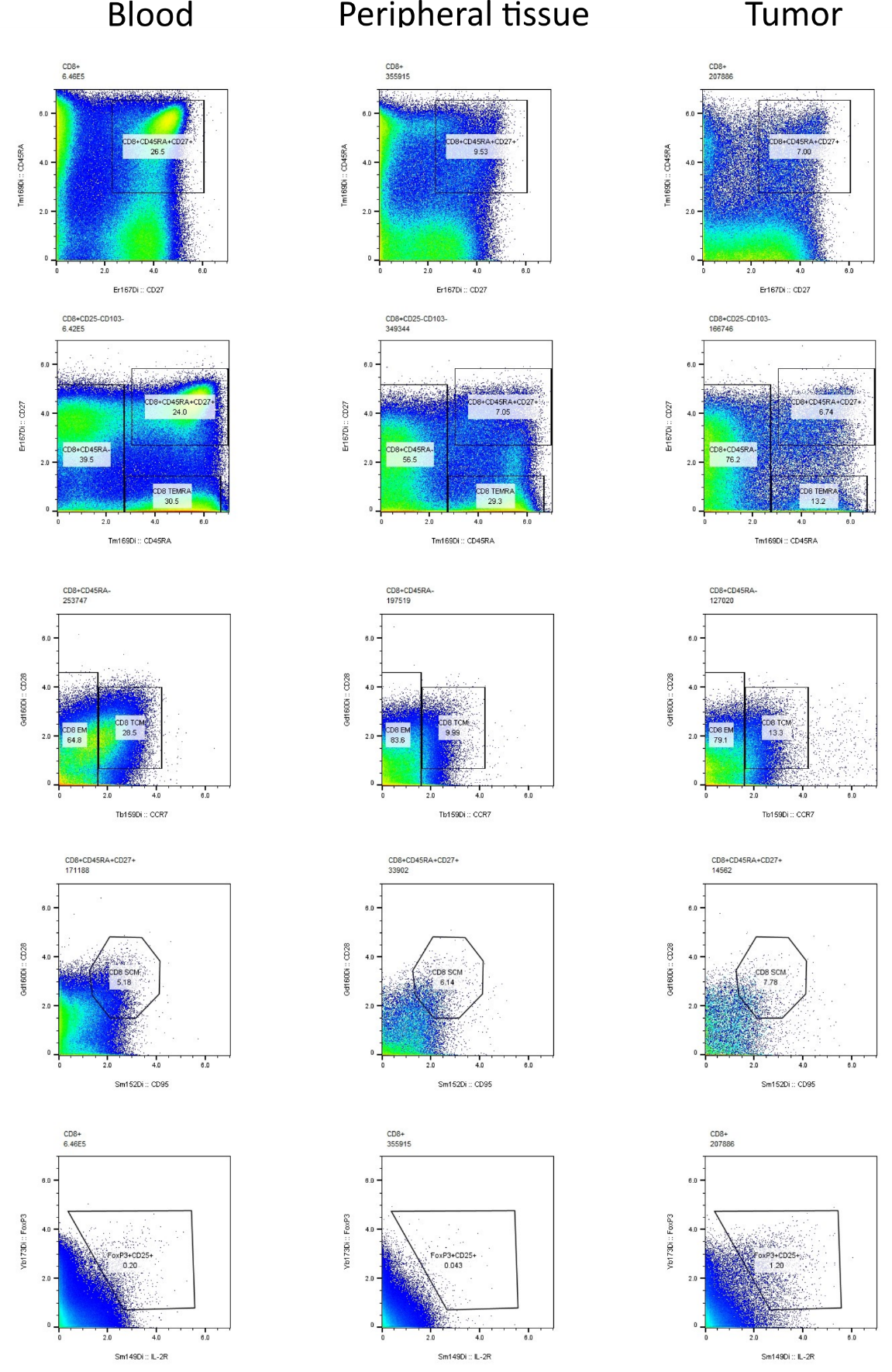


Figure S3. Manual gating of T cells

Manual gating of T cells shown for all CD45 positive cells. Shown are gates based on the same markers used to identify subtypes in unsupervised clustering. Percentage of cells within a specific gate are indicated. Expression values are scaled using arcsinh transformation.


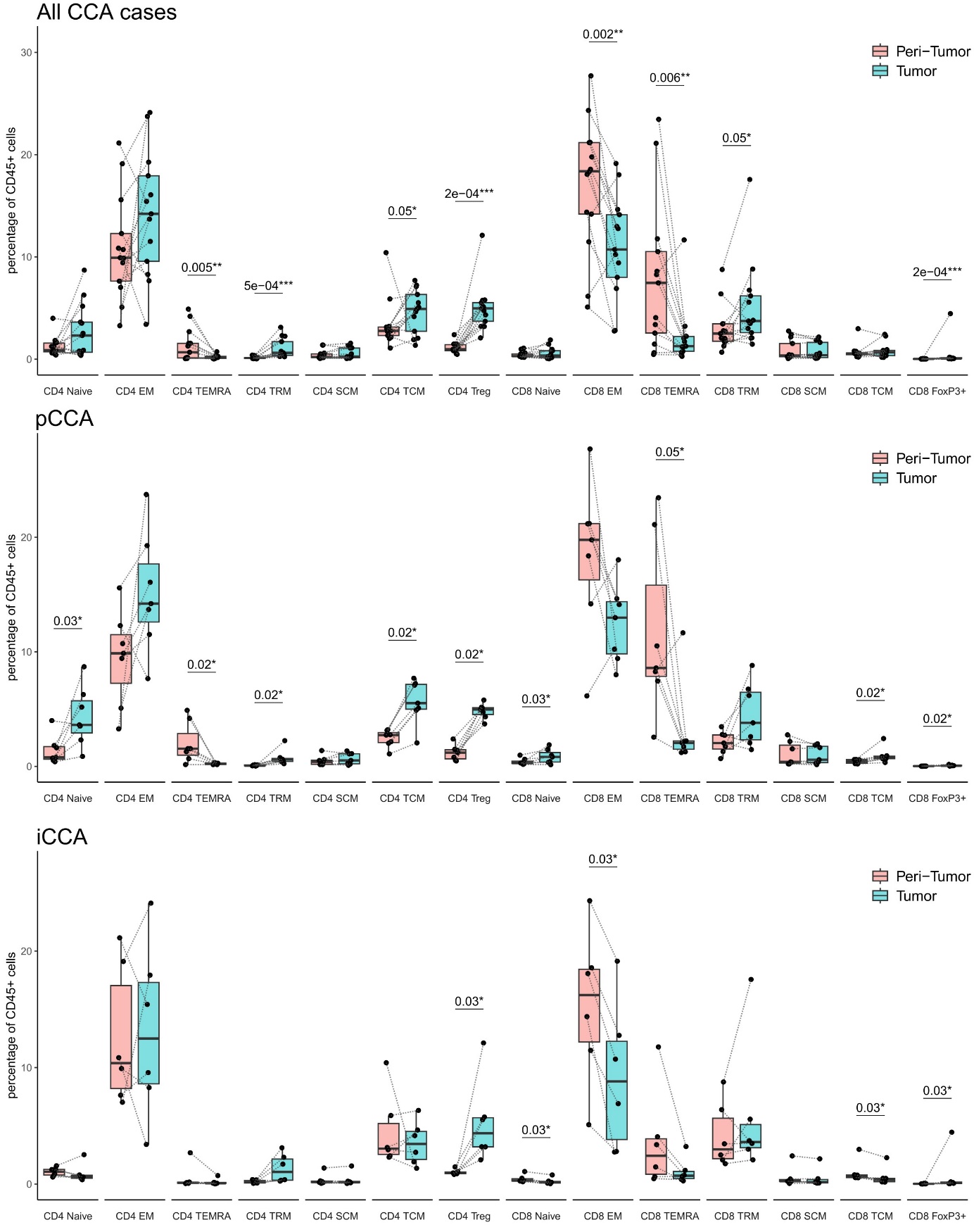


Figure S4. Analysis of T cell subsets across entities

T cells were analyzed using CyTOF. Subtypes were identified by annotation of unsupervised clusters. Abundances were compared between central tumor samples and samples from peripheral tissue (Peri-Tumor). Analysis was done for all CCA patients and pCCA and iCCA cases separately.


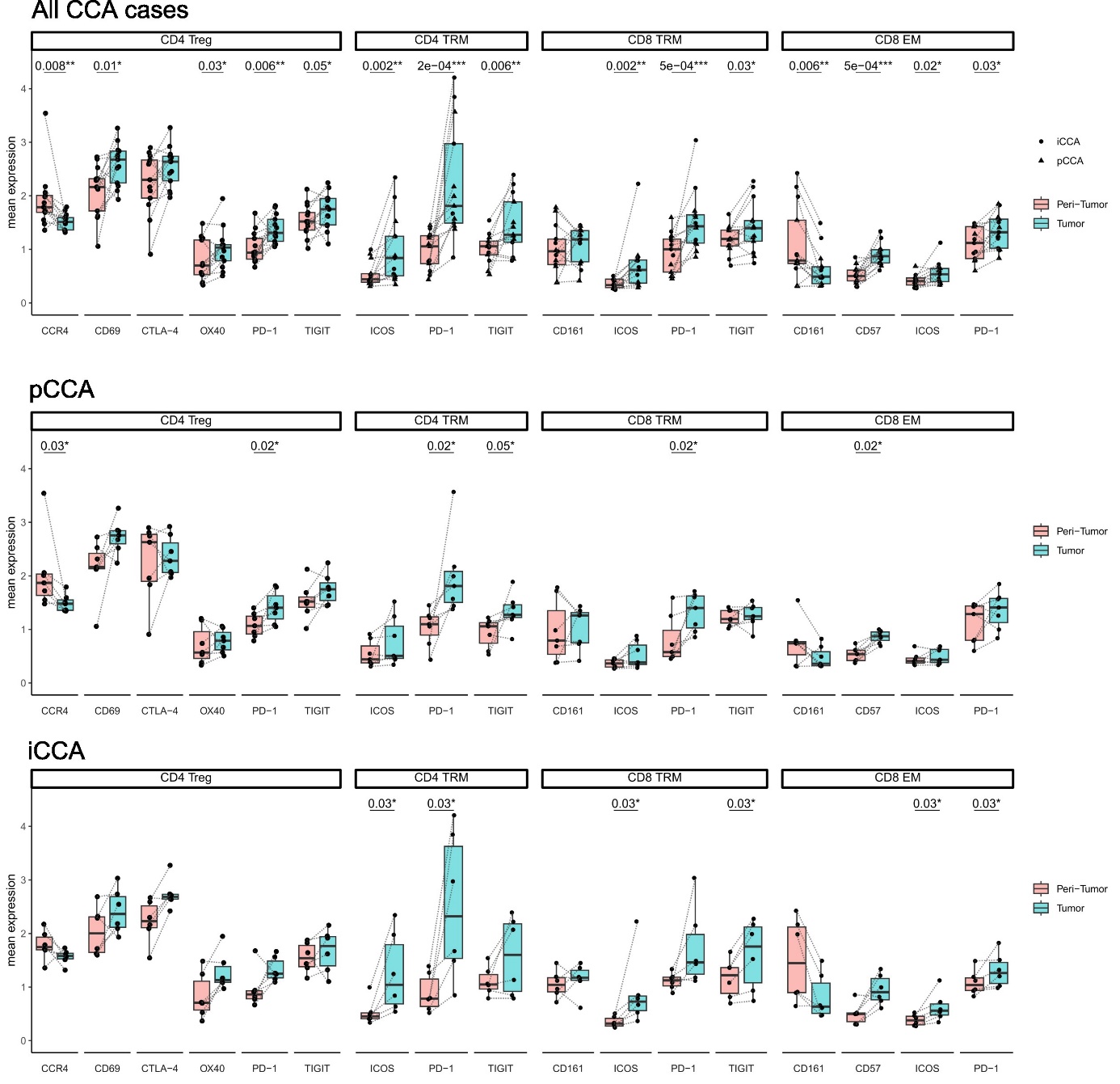


Figure S5. Analysis of marker expression across entities

Expression of key markers in CyTOF data within subsets of interest were compared between cells isolated from the central tumor and peripheral tumor (Peri-Tumor). Analysis was done for all CCA patients and pCCA and iCCA cases separately. Shown values are scaled using arcsinh transformation.

Table 2. Summary of changes in subtype abundance

Observed increase or decrease of subsets in the CyTOF cohort when comparing tumor against peri-tumor regions. Results are summarized for pCCA and iCCA cases combined (All CCA) and for iCCA and pCCA separately. Significance levels are indicated (n.s. = not significant, * p-value < 0.05, ** p-value < 0.01, *** p-value < 0.001)

| **Subset** | **Markers** | **All CCA** | | **pCCA** | | **iCCA** | |
| --- | --- | --- | --- | --- | --- | --- | --- |
|  |  | **Trend in CD4** | **Trend in CD8** | **Trend in CD4** | **Trend in CD8** | **Trend in CD4** | **Trend in CD8** |
| Naive | CD45RA+ CD27+ CCR7+ | Increase n.s. | Unchanged n.s. | Increased  * | Increased  * | Decreased n.s. | Decreased * |
| EM | CD45RA- CCR7- | Increased n.s. | Decreased ** | Increased n.s. | Decreased n.s. | Unchanged n.s. | Decreased n.s. |
| TEMRA | CD27- CD45RA+ | Decreased ** | Decreased ** | Decreased * | Decreased * | Unchanged n.s. | Decreased n.s. |
| TRM | CD103+ CD69+ | Increased *** | Increased  * | Increased  * | Increased n.s. | Increased n.s. | Increased n.s. |
| SCM | CD45RA+ CD27+ CD28+ CD95+ | Unchanged n.s. | Unchanged n.s. | Unchanged n.s. | Unchanged n.s. | Unchanged n.s. | Unchanged n.s. |
| TCM | CD45RA- CD28+ CCR7+ | Increased  * | Unchanged n.s. | Increased  * | Increased  * | Unchanged n.s. | Decreased * |
| Treg | IL-7R- IL2R+ FoxP3+ | Increased *** |  | Increased  * |  | Increased  * |  |


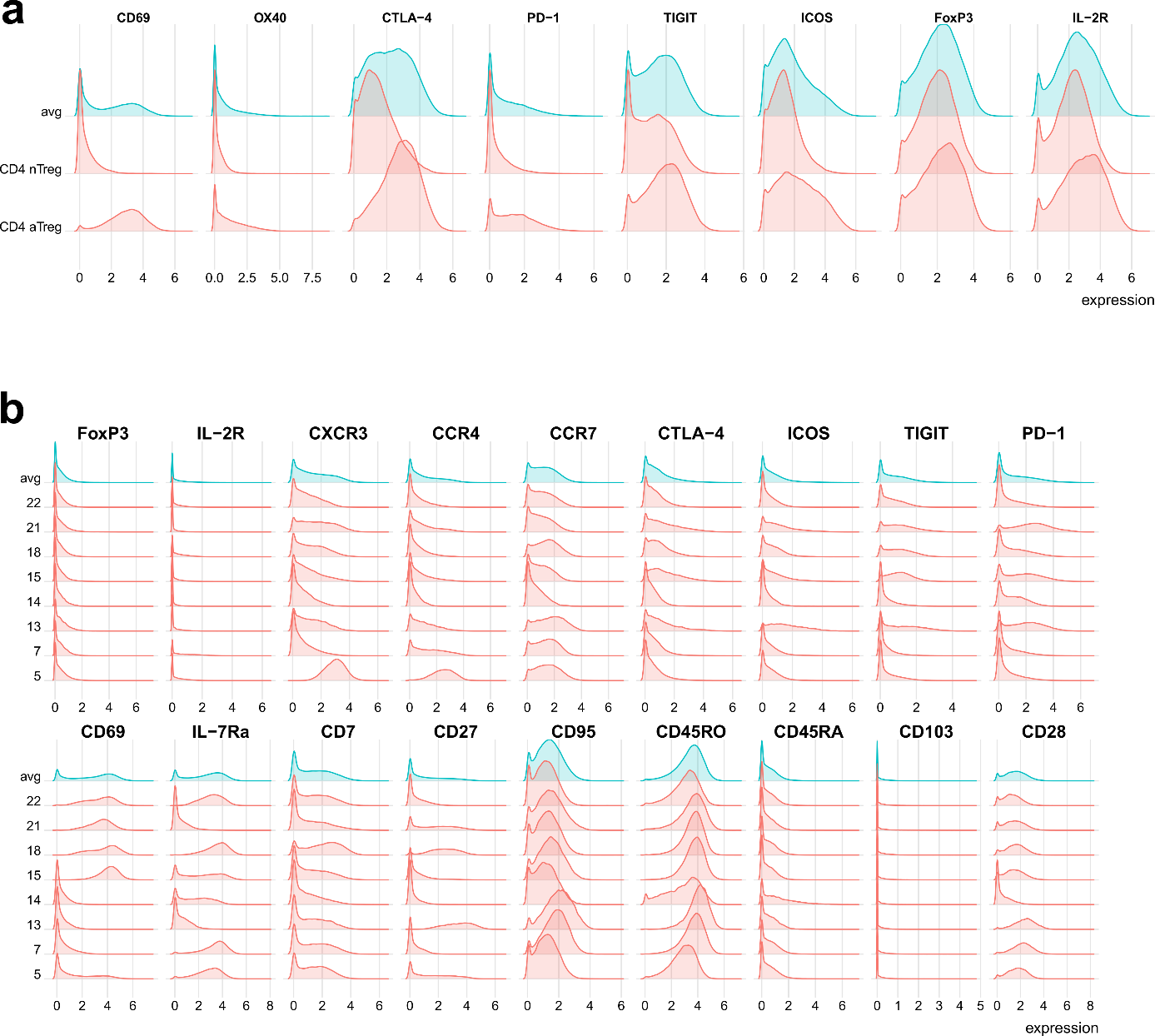


Figure S6. Marker expression of activated Tregs

**(a)** We identified two different clusters of regulatory CD4+ cells and based on their mean marker expression we annotated them into aTregs=activated Tregs and nTregs= non-activated Tregs. Shown are density plots of normalized marker expression. The upper column at the top shows the average expression across all identified Tregs. **(b)** Marker expression of all identified CD4 EM clusters. We observed an increased abundance of cluster 7 in the periphery for patients without lymph node metastasis.

Table 3. molecular subtypes of tumors determined by next-generation sequencing (NGS)

| Entity | Included in: | Detected variants per patient in gene: |
| --- | --- | --- |
| iCCA | CyTOF cohort | **Deletion:**  BAP1  **Single nucleotide variant:**  IDH1 (394C>T)  **Unknown significance:**  NOTCH1 |
| iCCA | CyTOF cohort | **Translocation:**  FGFR2 |
| pCCA | snRNA cohort (poor) | **Activating:**  KRAS (35G>T)  **Deletion:**  SMAD4  **Unknown significance:**  ERBB2, ATM |
| pCCA | CyTOF cohort  snRNA cohort (poor) | **Deletion**:  RB, SMARCA4  **Unknown significance:**  CDKN2A, CDKN2B, ATR, CCNE1 |
| pCCA | CyTOF cohort  snRNA cohort (good) | **Single nucleotide variant:**  KRAS (35 G>A)  **Unknown significance:**  IDH1, EPHB1, FANCA, KDM5C, MYC, ZNF703 |

Next-generation sequencing data was available for 3 of the pCCA and 2 of the iCCA patients included in the CyTOF and snRNA seq cohorts.


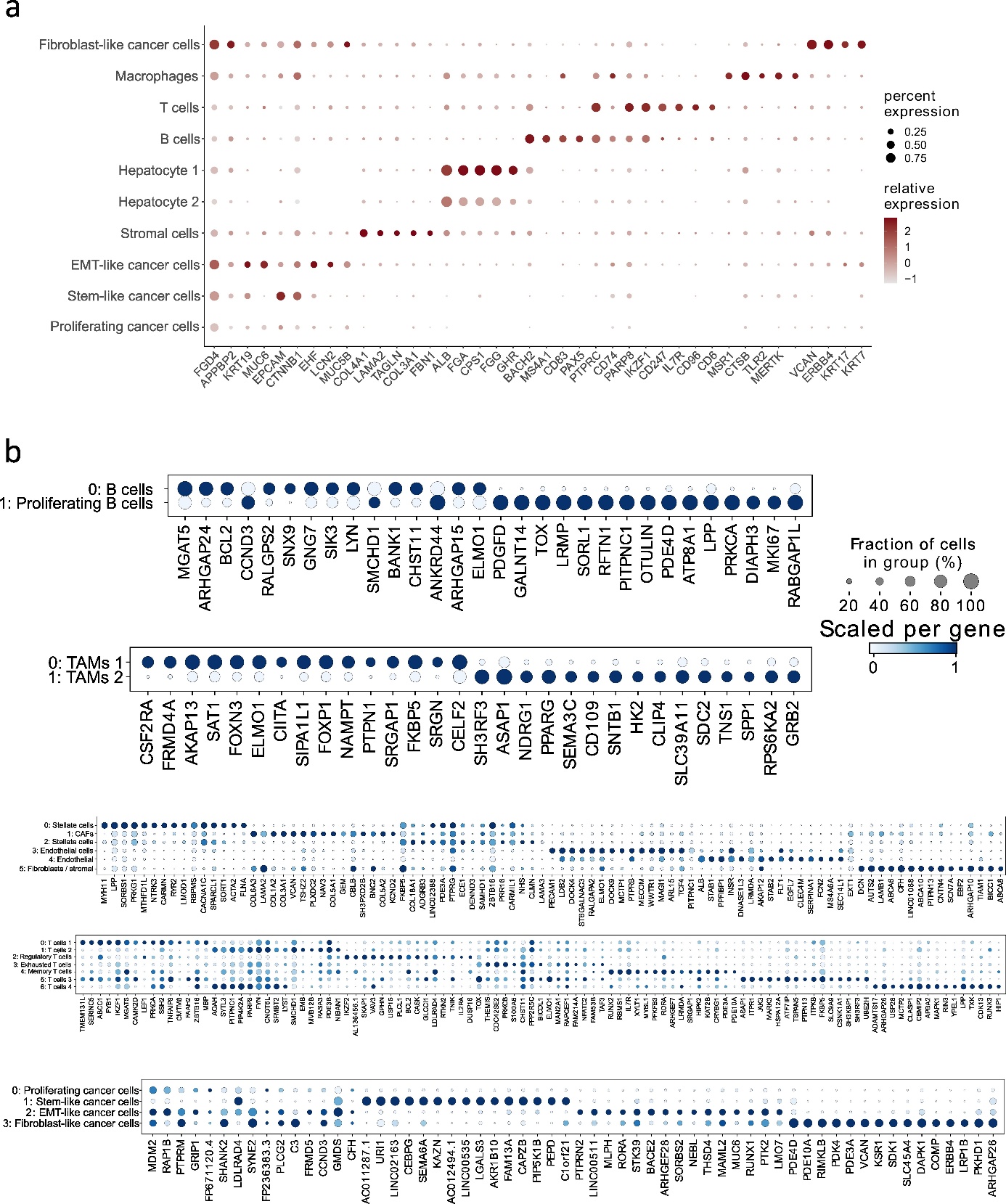


Figure S7. snRNA maker expression of clusters and subclusters

Marker gene expression of cells identified in snRNA sequencing data. **(a)** Cells were clustered into 9 cell types. 4 of them were identified as cancer cells and annotated based on expression of functional genes. (**b**) B cells, Macrophages, stromal cells and T cells were subclustered and further annotated. Marker gene expression of the previously identified 4 cancer subsets compared to the total cancer population was used to further describe them.


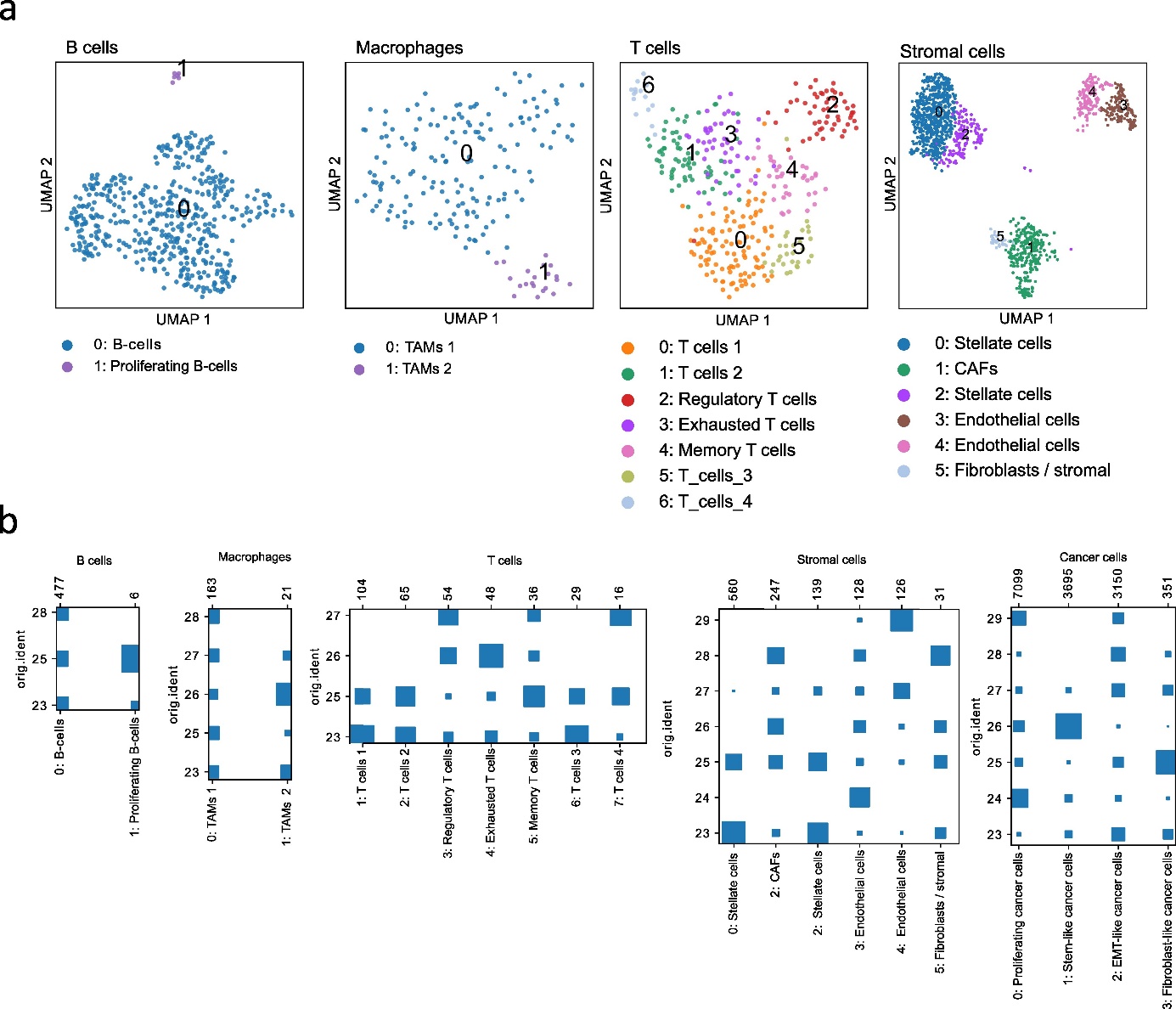


Figure S8. Subcluster abundance

Previously identified cell types were subclustered. (**a**) UMAP of subclustering results overlayed with the annotation. (b) Number of cells per subcluster across patients.


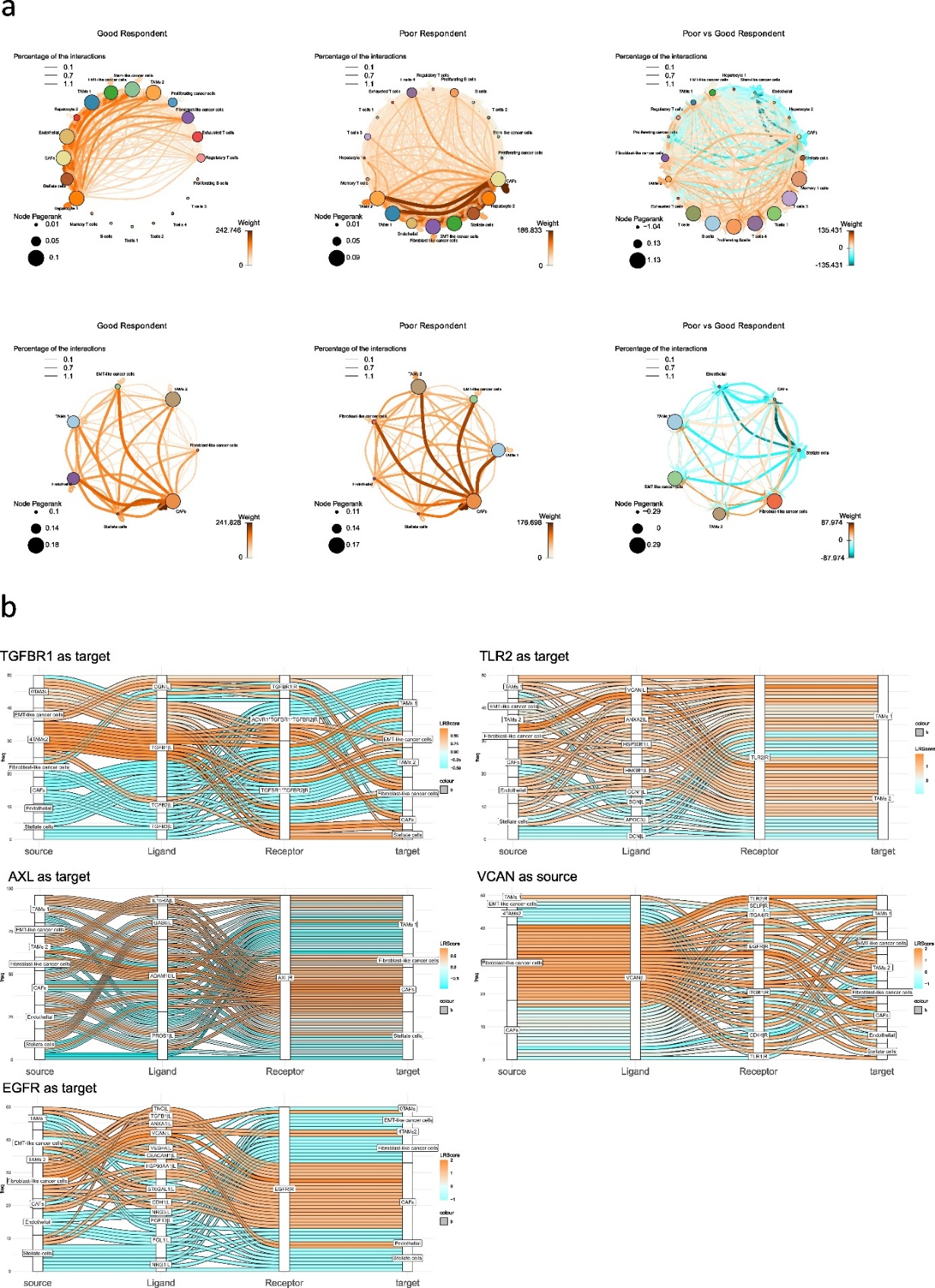


Figure S9. Cell-cell communication

Expression of ligand-receptor pairs was used to infer communication between the identified clusters. (**a**) Network graph depicting comparative cell-cell communication plotted for good response patients (left), poor prognosis patients (middle) and comparative plot between the prognosis groups (right). Network graphs were plotted for all identified clusters (top) and for clusters most involved in the cell-cell communication (bottom). **(b)** Sankey plots visualize interactions of some of the top ligands and receptors comparing good and poor prognosis. Shown are interactions of TGFBR1, TLR2, Axl and EGFR as target receptors and VCAN as source ligand.


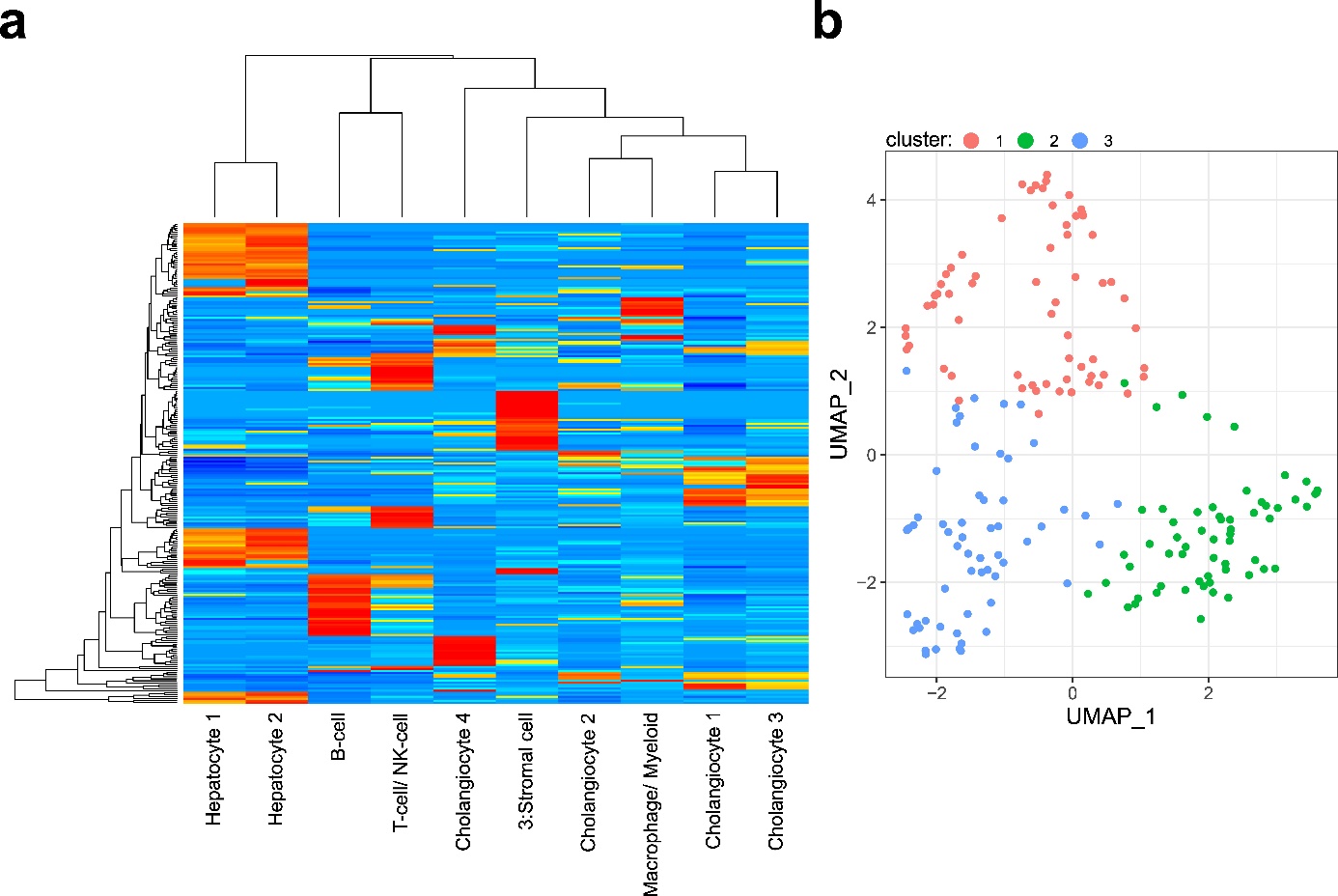


Figure S10. Signature matrix is used for cellular deconvolution and clustering based on the deconvolution results

**(a)** We used cibersortx to construct a signature matrix based on our 10 clusters identified in the snRNA data. The signature matrix was used to predict cellular fractions in public transcriptomic data from 182 eCCA patients. **(b)** Relative fractions within the samples were used as input for dimensionality reduction and clustering resulting in 3 distinct clusters.

Table 4. Number of cells acquired in CyTOF cohort. Samples with more than 50000 CD4 or CD8 cells were donwsampled to only contrivbute 50000 cells to the analysis

| **Patient** | **Tissue** | **CD4** | **CD8** | **Miscellaneous** | **CD45+ cells** |
| --- | --- | --- | --- | --- | --- |
| 304 | Blood | 31134 | 7467 | 100936 | 139537 |
|  | Peripheral tissue | 27684 | 21482 | 65867 | 115033 |
|  | Tumor | 5645 | 4355 | 55895 | 65895 |
| 307 | Blood | 121581 | 41161 | 68459 | 231201 |
|  | Peripheral tissue | 7906 | 7905 | 14389 | 30200 |
|  | Tumor | 3352 | 2233 | 8277 | 13862 |
| 309 | Blood | 70077 | 25474 | 119149 | 214700 |
|  | Peripheral tissue | 9052 | 6742 | 53135 | 68929 |
|  | Tumor | 17226 | 7427 | 55221 | 79874 |
| 320 | Blood | 56545 | 17997 | 72758 | 147300 |
|  | Peripheral tissue | 537 | 662 | 9284 | 10483 |
|  | Tumor | 13429 | 13600 | 29660 | 56689 |
| 321 | Blood | 121809 | 57859 | 181908 | 361576 |
|  | Peripheral tissue | 4469 | 4680 | 24947 | 34096 |
|  | Tumor | 31514 | 16797 | 26203 | 74514 |
| 323 | Blood | 6974 | 4437 | 121487 | 132898 |
|  | Peripheral tissue | 3182 | 2974 | 31038 | 37194 |
|  | Tumor | 3863 | 2277 | 17055 | 23195 |
| 325 | Blood | 70949 | 39326 | 109910 | 220185 |
|  | Peripheral tissue | 14583 | 38202 | 47886 | 100671 |
|  | Tumor | 28252 | 49991 | 39109 | 117352 |
| 326 | Blood | 60028 | 22074 | 65393 | 147495 |
|  | Peripheral tissue | 1963 | 3220 | 10821 | 16004 |
|  | Tumor | 34947 | 13854 | 62371 | 111172 |
| 329 | Blood | 60940 | 54287 | 72844 | 188071 |
|  | Peripheral tissue | 13417 | 28627 | 19490 | 61534 |
|  | Tumor | 11533 | 7183 | 13176 | 31892 |
| 331 | Blood | 99459 | 45144 | 104471 | 249074 |
|  | Peripheral tissue | 24403 | 32907 | 50373 | 107683 |
|  | Tumor | 29831 | 33839 | 65311 | 128981 |
| 332 | Blood | 72461 | 24260 | 180032 | 276753 |
|  | Peripheral tissue | 21555 | 58548 | 92003 | 172106 |
|  | Tumor | 28707 | 20844 | 44550 | 94101 |
| 333 | Blood | 206002 | 209994 | 215128 | 631124 |
|  | Peripheral tissue | 43584 | 52914 | 40561 | 137059 |
|  | Tumor | 25208 | 15162 | 24549 | 64919 |
| 336 | Blood | 55332 | 54167 | 98670 | 208169 |
|  | Peripheral tissue | 11048 | 37024 | 42073 | 90145 |
|  | Tumor | 12175 | 8130 | 37459 | 57764 |
| 337 | Blood | 70799 | 15971 | 96262 | 183032 |
|  | Peripheral tissue | 14433 | 17936 | 38590 | 70959 |
|  | Tumor | 13870 | 12979 | 15849 | 42698 |
| 352 | Blood | 115681 | 113204 | 210270 | 439155 |
|  | Peripheral tissue | 37284 | 103911 | 76499 | 217694 |
|  | Tumor | 43090 | 28301 | 48444 | 119835 |

Table 5. Antibodies, reagents, and dyes used for immunohistochemistry.

| **Antibody/ dye/ reagent** | **Dilution** | **Source** | **Identifier** |
| --- | --- | --- | --- |
| Recombinant anti-cytokeratin 19 antibody | 1:500 in PBS | Abcam | ab52625 |
| Monoclonal Mouse Anti-Human CD68 | 1:500 in PBS | Agilent Dako | M0876 |
| Axl Polyclonal Antibody | 1:500 in PBS | Thermo Fisher | PA5-77875 |
| GAS6 Polyclonal Antibody | 1:500 in PBS | Thermo Fisher | PA5-79300 |
| TLR2 Polyclonal Antibody | 1:250 in PBS | Thermo Fisher | PA1-21611 |
| Versican Recombinant Rabbit Monoclonal Antibody (6D8K9) | 1:500 in PBS | Thermo Fisher | MA5-42721 |
| EGFR Monoclonal Antibody (H11) | 1:500 in PBS | Thermo Fisher | MA5-13070 |
| Anti-TGF beta 1 antibody [EPR21143] | 1:500 in PBS | Abcam | ab215715 |
| Ultra-Map anti rabbit HRP | Ready to use | Roche |  |
| DISC. UltraMap anti-Goat multimer RUO | Ready to use | Roche | 6607241001 |
| DISCOVERY OmniMap anti-Ms HRP (RUO) | Ready to use | Roche | 5269652001 |
| Chromo Map DAB | Ready to use | Roche | 5266645001 |
| DISCOVERY Teal HRP Kit (RUO) | Ready to use | Roche | 8254338001 |
| DISC. Purple Kit RUO | Ready to use | Roche | 7053983001 |
| Hematoxylin II | Ready to use | Roche | 05 277 965 001 |
| Bluing Reagent | Ready to use | Roche | 05 266 769 001 |
